# Multi-subject hierarchical inverse covariance modelling improves estimation of functional brain networks

**DOI:** 10.1016/j.neuroimage.2018.04.077

**Published:** 2018-09

**Authors:** Giles L. Colclough, Mark W. Woolrich, Samuel J. Harrison, Pedro A. Rojas López, Pedro A. Valdes-Sosa, Stephen M. Smith

**Affiliations:** a Oxford Centre for Human Brain Activity (OHBA), Wellcome Centre for Integrative Neuroimaging, Department of Psychiatry, University of Oxford, Oxford, UK; b Oxford Centre for Functional MRI of the Brain (FMRIB), Wellcome Centre for Integrative Neuroimaging, Nuffield Department of Clinical Neurosciences, University of Oxford, Oxford, UK; c Centre for Doctoral Training in Healthcare Innovation, Institute of Biomedical Engineering Science, Department of Engineering, University of Oxford, Oxford, UK; d Neuroinformatics Department, El Centro de Neurociencias de Cuba (CNEURO), La Habana, Cuba; e The Clinical Hospital of Chengdu Brain Science Institute, MOE Key Lab for Neuroinformation, University of Electronic Science and Technology of China, Chengdu, China

**Keywords:** fMRI, MEG, Functional connectivity, Gaussian Graphical models, Hierarchical Bayesian models, Concentration graph, Precision model, Inverse covariance model, MCMC

## Abstract

A Bayesian model for sparse, hierarchical, inver-covariance estimation is presented, and applied to multi-subject functional connectivity estimation in the human brain. It enables simultaneous inference of the strength of connectivity between brain regions at both subject and population level, and is applicable to fMRI, MEG and EEG data. Two versions of the model can encourage sparse connectivity, either using continuous priors to suppress irrelevant connections, or using an explicit description of the network structure to estimate the connection probability between each pair of regions. A large evaluation of this model, and thirteen methods that represent the state of the art of inverse covariance modelling, is conducted using both simulated and resting-state functional imaging datasets. Our novel Bayesian approach has similar performance to the best extant alternative, Ng et al.'s Sparse Group Gaussian Graphical Model algorithm, which also is based on a hierarchical structure. Using data from the Human Connectome Project, we show that these hierarchical models are able to reduce the measurement error in MEG beta-band functional networks by 10%, producing concomitant increases in estimates of the genetic influence on functional connectivity.

## Introduction

The estimation of functional connectivity in the human brain (Friston, 2011; Smith et al., 2013) is becoming a key tool forenhancing our understanding of disease and cognition as part of functional magnetic resonance imaging (fMRI) and magnetoencephalography (MEG) studies. The most important and exciting uses of this type of analysis focus on individual differences in connectivity patterns. Subjects' functional connectomes are heritable (Colclough et al., 2017; Glahn et al., 2010); are associated with cognitive ability (Finn et al., 2015), and with wealth, health and life satisfaction (Smith et al., 2015); provide neuromarkers for sustained attention (Rosenberg et al., 2016); are implicated with a range of diseases and disorders (Greicius, 2008; Stam, 2014); and predict task-evoked activity (Tavor et al., 2016). For all of these forms of analysis, accurate estimation of single-subject functional networks is crucial.

Despite the recent explosion of research and high-quality findings, whole-brain functional connectivity estimation is relatively immature. Most of the key developments highlighted above use very simple Gaussian graphical models (GGMs) for the covariance of the data, in which the partial correlations between regions indicate the strengths of connections. We focus on this approach. However, accurate estimation of individual subjects' functional networks using GGMs can be difficult, particularly without long acquisition times. In an effort to improve the accuracy of network estimation, sparsity in the networks tends to be encouraged by suppressing weak connections (Dempster, 1972; Duff et al., 2013; Smith et al., 2013; Varoquaux and Craddock, 2013). Imposition of sparsity can also aid interpretation, by explicitly suggesting that certain individual functional connections are absent. There is even an entire field that attempts to characterise the function and dysfunction of cognitive networks using certain properties of this underlying graph structure (Bullmore and Sporns, 2009; de Pasquale et al., 2012, 2015; Stam and van Straaten, 2012; van Straaten and Stam, 2013).

While considerable work has been expended upon sparse network estimation for individual datasets (Friedman et al., 2008; Hinne et al., 2014, 2015; Lenkoski, 2013; Mazumder and Hastie, 2012a,b; Mohammadi and Wit, 2015; Ryali et al., 2012; Wang, 2012a,b, 2015), relatively little effort has been made towards the joint inverse covariance estimation relevant for multi-subject, whole-brain network inference (Our most complete list is Danaher et al., 2015; Guo et al., 2011; Harrison et al., 2015; Lee and Liu, 2015; Liang et al., 2016; Marrelec et al., 2006; Mejia et al., 2018; Ng et al., 2013; Peterson et al., 2015; Qiu et al., 2015; Varoquaux et al., 2010; and Yang et al., 2015. Also of note is the work of Nadkarni et al., 2017, who fit multiple Gaussian networks under auto-regressive processes to model MEG data, and Hinne et al., who in 2013 developed a hierarchical connectivity model for structural brain networks inferred from diffusion MRI data.). Models with a ‘hierarchical’ structure, which simultaneously estimate the population connectivity and each individual's network strengths, should improve the quality of inference (Gelman et al., 2014; Woolrich, 2008). Some of the existing methods attempt a hierarchical model for the structure of the network, so that the probability of a connection existing in each subject is influenced by the group's connection map. When it comes to the connection *strengths*, only Ng et al. model the relationship between subject and group-level connectivities within their penalised maximum-likelihood approach. None combine sparse network priors, a hierarchical design that shares information on the strengths of connections over the whole dataset, and a computationally-efficient Bayesian inference framework that can be applied to large multi-subject neuroimaging datasets.

We present a new hierarchical model and scalable inference framework for sparse Bayesian modelling of multiple inverse covariance matrices. It is applied to the estimation of functional brain networks, with joint characterisation of subject-level and population-average connectivities. We model functional connectivity simply as undirected partial correlations between the network nodes—this model can be applied to MEG data (Colclough et al., 2015) in addition to fMRI, and is among the most successful and repeatable of measures in either modality (Colclough et al., 2016; Ramsey et al., 2014; Smith et al., 2011). We show that the posterior can be reformulated as a series of linked linear regressions, allowing a broad class of sparse priors to be applied to covariance modelling. Two particular priors are compared. The first imposes an explicit shared sparsity structure on the network graph, producing a posterior distribution over the edges present in the network. The second uses continuous priors to regularise the group connection strengths, more weakly encouraging network sparsity. A custom Markov chain Monte Carlo (MCMC) approach is used for inference, and we characterise how the computation time scales with model dimension and the number of subjects.

We run a large evaluation of the performance of our model and the current state of the art in GGM estimation. This evaluation uses simulated data to test models' ability to reconstruct connection strengths and sparse network patterns. We also use truncated segments of resting-state fMRI and MEG recordings from the Human Connectome Project (HCP) to assess inference quality with very short or noisy datasets. Finally, we use trait prediction analyses from the fMRI networks and genetic influence analyses on the MEG networks to demonstrate noise reductions when subject and population connectivities are estimated with a hierarchical framework.

We start with an overview of our new Bayesian model and inference approach.

## A hierarchical model for inverse covariance matrices

In order to jointly estimate connectivity over many subjects, we need a scalable covariance inference framework that can be formulated as a hierarchical model. Most existing Bayesian models for GGMs use *G*-Wishart priors (Letac and Massam, 2007). These are challenging to incorporate into a hierarchy because of the difficulty in computing the normalising constant of the distribution, itself a function of the underlying graph structure. Trans-dimensional MCMC approaches that avoid this computation have been developed for models of single covariance matrices (Hinne et al., 2014, 2015; Lenkoski, 2013; Mohammadi and Wit, 2015; Wang, 2012b), and an analytic expression for the troublesome normalising constant has been recently proposed (Uhler et al., 2018), but building a sampler for multiple *G*-Wishart distributions with an inferred group prior and shared graph structure is not trivial.

Instead, we take a different approach, inspired by an alternative prior structure. Wang describes, in 2012*a* and 2015, two different priors that allow simple block-Gibbs sampling along the columns of matrices to draw from the posterior of two specific models for covariance. We build on this idea, by demonstrating that the conditional distribution of one column of a precision matrix takes the form of a linear regression, and that this reformulation gives access to most of the existing priors and inference engines from the Bayesian linear regression literature, enabling a range of hierarchical GGM models to be implemented.

Like most other Bayesian GGM or covariance models, we build sparse priors for the precision (or inverse covariance) matrix. Dempster argued in 1972 that introducing sparsity to the precision, rather than the covariance matrix, was the more desirable option, because this choice maximises the entropy of the resulting distribution. It also makes for a more interpretable approach, as promotion of sparsity in the precision or partial correlation matrix can be directly understood as promoting sparsity in the underlying GGM. The zeros in the partial correlation matrix directly indicate the lack of an edge in the network. Additionally, in our application of functional connectivity estimation, previous studies suggest that partial correlations derived from the precision matrix may be more robust network estimators than full correlations, particularly if there are sufficient data to make good estimates (Duff et al., 2013; Marrelec et al., 2006; Smith et al., 2011). In his 2015 paper, Wang designed an additional prior that imposes sparsity in the covariance, not the precision matrix. Our extension of his prior for precisions could be easily adapted for sparse covariance matrices if desired.

The models we propose have three principal features. First, the connection strengths of each subject, for a particular network edge, are distributed with some variance about the population connectivity strength. This regularises subjects towards the group mean, in a similar fashion to the $L_{2}$ penalty used in Ng et al. (2013). Second, the population connectivity is constrained using a Cauchy prior (Polson and Scott, 2012), which has a large mass near zero. This prior has many similarities to the double-exponential prior distribution, which has the same form as the widely-used $L_{1}$ penalty for sparsity promotion (Danaher et al., 2015; Friedman et al., 2008; Ng et al., 2013; Varoquaux et al., 2010; Wang, 2012a). These two features alone create a sparse, hierarchical inverse covariance model. We form a second model by adding a final feature that regularises using an explicit sparse network structure. The probability of each network connection being present or absent is directly inferred using a spike and slab prior (Mitchell and Beauchamp, 1988). This strong sparsity modelling is a feature of the Bayesian approach, and is not possible to frame as a convex optimisation problem.

Below, we set out the likelihood of the region of interest (ROI) data in each subject. Then we reformulate the inference of inverse covariance matrices as a linear regression problem under a broad range of priors, and position our two forms of the hierarchical model within this framework. Full details of our inference program and MCMC algorithm for this model, denoted HIPPO (Hierarchical Inference of Posterior Precisions in OSL^1^), are given in the supplementary material.

### Likelihood for the connectivity model

We describe the (temporally demeaned) activations $Y_{s}=\left[y_{1}^{s},y_{2}^{s},\ldots y_{n_{s}}^{s}\right]\in {\mathbb{R}}^{p\times n_{s}}$, within *p* ROIs, sampled at $n_{s}$ time points, for each subject *s*, as being drawn independently from a multivariate Gaussian distribution with zero mean and precision matrix $\Omega_{s}$

(1) $$Y_{s}∼N\left(0,\;\Omega_{s}^{-1}\right),\;\Omega_{s}\in {\mathbb{P}}_{G},$$

where ${\mathbb{P}}_{G}$ is the cone of positive definite p×p matrices restricted to the graph G=(V,E) such that an absence of an edge from set *E* implies conditional independence of the two relevant variables in all subjects,

$$\left(i,j\right)\notin E\Rightarrow \omega_{ij}^{s}=0\;\forall \;s\text{.}$$

We use the general term *activation* to encompass changes in blood-oxygenation-level dependent (BOLD) response over time in fMRI, or fluctuations in the power envelope of oscillatory activity measured with MEG or electroencephalography (EEG).

### Precision modelling as linked linear regression

Building on the work in Wang, 2012a, Wang, 2015, we show that a very broad range of priors from the linear regression literature can be applied to the elements of a precision matrix, with a simple restriction on the prior for the diagonal elements. Inference can be performed as a series of draws from the conditional distributions of linked columns of variables over all subjects. As long as the prior factorises over the elements of the precision matrix, it is possible to introduce layers of hyper-parameters without breaking this sampling approach. This will enable us to build a large hierarchical model within a tractable sampling framework.

Some notation is useful. We partition subjects' precision matrices as follows,

(2) $$\Omega_{s}=\left(\begin{array}{cc} \Omega_{11}^{s} & \omega_{12}^{sT}\\ \omega_{12}^{s} & \omega_{22}^{s} \end{array}\right)\;\text{.}$$

Without loss of generality, we can discuss just the final column of precision matrix $\Omega_{s}$, ${\left[\omega_{12}^{s},\omega_{22}^{s}\right]}^{T}$. Let $\Omega_{11}^{s}$ represent the first principal minor (the block matrix without the final row or column), † the conjugate transpose operator, and let $S=Y_{s}^{†}Y_{s}=n_{s}\Sigma_{s}$ be the sample inner product matrix of subject *s*. Similar subscripts indicate identical partitions of other matrices, so for an inner product matrix, $S_{22}$ is the diagonal element of the selected column and $S_{12}$ the off-diagonal elements of the column. This is the same convention employed in Friedman et al.'s exposition of the graphical least absolute shrinkage and selection operator (LASSO) in 2008.

We define independent exponential prior distributions on the diagonal elements of the precision matrices, and require the priors on the off-diagonal elements to factorise over the elements (although in addition to any hyper-parameter matrices, Ψ, such as group-level connectivity strengths, they may share some common scalar hyper-parameters, θ),

(3) $$\pi \left(\Omega_{s}\right)=\prod_{i=1}^{p}Exp\left(\omega_{ii}^{s};\;\frac{\lambda^{s}}{2}\right)\prod_{i<j}\pi \left(\omega_{ij}^{s};\;\psi_{ij},\theta \right)\pi \left(\psi_{ij};\;\theta \right)\text{,}$$

using π(⋅) to denote a prior probability density. Combining (1) and (3), we can extract the conditional posterior for a column of the precision matrix,

(4) $$\begin{array}{ll} p\left(\omega_{12}^{s},\omega_{22}^{s},\psi_{12}|-\right) & \propto \;\frac{1}{{\left(2\pi \right)}^{\frac{n_{s}}{2}}}{\left(\omega_{22}^{s}-\omega_{12}^{s†}\Omega_{11}^{s-1}\omega_{12}^{s}\right)}^{\frac{n_{s}}{2}}{\left|\Omega_{11}^{s-1}\right|}^{-\frac{n_{s}}{2}}\\ & \times \exp\left(-\frac{1}{2}\left[S_{22}\omega_{22}^{s}+2S_{12}^{†}\omega_{12}^{s}\right]\right)Exp\left(\omega_{22}^{s};\;\frac{\lambda^{s}}{2}\right)\pi \left(\omega_{12}^{s};\;\psi_{12},\theta \right)\pi \left(\psi_{12};\;\theta \right)\;. \end{array}$$

Performing the variable substitution (Wang, 2012a)

(5) $$\left(u^{s},\;\nu^{s}\right)=\left(\omega_{12}^{s},\;\omega_{22}^{s}-\omega_{12}^{s†}\Omega_{11}^{s-1}\omega_{12}^{s}\right)\;\text{,}$$

for which the Jacobian is the identity matrix, we obtain

(6) $$\begin{array}{lll} \log\;p\left(u^{s},\nu^{s},\psi_{12}|-\right) & = & \frac{n_{s}}{2}\text{log}\nu^{s}-\frac{1}{2}\left(S_{22}+\lambda^{s}\right)\nu^{s}\\ & & -\frac{1}{2}u^{s†}\left[\left(S_{22}+\lambda^{s}\right)\Omega_{11}^{s-1}\right]u^{s}-S_{12}^{†}u^{s}\\ & & +\log\;\pi \left(u^{s};\;\psi_{12},\theta \right)+\log\;\pi \left(\psi_{12};\;\theta \right)+const\text{.} \end{array}$$

We can tidy up with the substitution ${ϒ}^{s}=\left(s_{22}+\lambda^{s}\right)\Omega_{11}^{s-1}$ to give a normal form for $u^{s}$ and a Gamma distribution on $\nu^{s}$,

(7) $$\log\;p\left(u^{s},\psi_{12}|-\right)=\;-\frac{1}{2}u_{s†}{ϒ}^{s}u^{s}-S_{12}^{†}u^{s}+\log\;\pi \left(u^{s};\;\psi_{12},\theta \right)+\log\;\pi \left(\psi_{12};\;\theta \right)-\frac{n_{s}}{2}\log\;\pi +const\text{.}$$

(8) $$\log\;p\left(\nu^{s}|-\right)=\;\frac{n_{s}}{2}\text{log}\nu^{s}-\frac{1}{2}\left(S_{22}+\lambda^{s}\right)\nu^{s}+const\text{.}$$

Equations (7), (8) provide a basic block-Gibbs sampling scheme in which all variables associated with a column, across all subjects, are drawn together. It is important that the sampled matrices are positive definite, to qualify as valid precision matrices. The design of this aspect of the sampling algorithm (as described for a single precision matrix by Wang) ensures this condition. If, on each draw of the variables within a column, the principal minors $\Omega_{11}^{s}$ are positive definite, then the updated matrices will by definition be positive definite if the Schur complement $\omega_{22}^{s}-\omega_{12}^{s†}\Omega_{11}^{s-1}\omega_{12}^{s}$ is greater than zero (Boyd and Vandenberghe, 2004). This inequality is enforced by the strictly positive Gamma distribution on $\nu^{s}$. Assuming the sampler is well initialised, the algorithm guarantees positive definite precision matrices on each and every update.

We draw the comparison to conventional linear regression, y=Xβ+ε, $\varepsilon ∼N\left(0,\sigma^{2}\right)$, conditional on $\sigma^{2}$,

$$\begin{array}{l} p\left(\beta |y,X,\sigma^{2}\right)\propto \frac{1}{{\left(2\pi \sigma^{2}\right)}^{\frac{n_{s}p}{2}}}e^{-\frac{1}{2}\beta^{†}ϒ\beta +r^{†}\beta }\;\pi \left(\beta ;\;\theta \right)\\ ϒ=\frac{X^{†}X}{\sigma^{2}}\\ r=\frac{y^{†}X}{\sigma^{2}}\text{,} \end{array}$$

to see that this factorisation into column-conditionals leads to inference as a set of *p* linked regressions on one variable and its interactions, given all the other variables. The link between partial correlation estimation and regression problems has been identified previously (Peng et al., 2009), but within this Bayesian inference context, the key point is that we can now borrow sparse priors from the extensive linear regression literature. We are free to choose any prior that can factorise over the off-diagonal elements of the precision matrix and retain the simple block-Gibbs sampling scheme (7) and (8). Moreover, we are free to build a hierarchy of prior distributions over the elements of the precision matrices, so long as priors factorise over $\omega_{ij}$ when conditioned on the hyper-parameters. Sampling is possible in this framework by alternating block-Gibbs draws of $p\left(u^{s},\psi_{12}\;|\;-\right)$, the conditional distribution of the columns of precision matrices within each subject $\omega_{12}^{s}$ and of hyper-parameter matrices $\psi_{12}$ (which might represent group-level connection strengths or a sparsity structure, for example), with Gibbs draws from the conditional distributions of any common hyper-parameters, $p\left(\theta \;|\;-\right)$.

### Hierarchical sparse priors for precision matrices

Using the framework developed above, we describe two Bayesian sparse hierarchical models for inverse covariance matrices. The first explicitly models the presence or absence of edges within the functional network, strongly promoting sparsity in the system. The second removes this feature, and relies on continuous priors on the group connection strengths to suppress weak edges towards zero.

#### Model 1: a strongly sparse prior

For each subject, we place an exponential prior on the diagonal elements of the precision matrix, as in (3). This choice allows us to implement the column-wise sampling scheme described in equations (4)–(8). The free parameter in this distribution, $\lambda^{s}$, is given (for each subject) the ‘neutral’ Gamma conjugate hyperprior (Kerman, 2011). There is normally plenty of information with which to estimate the diagonal elements (inverse variances), and so an uninformative prior is appropriate. The neutral Gamma prior is relatively uninformative on $\text{log}\lambda^{s}$ and is claimed to perform better than traditional Ga(ε,ε) priors (*ibid.*),

(9) $$\lambda^{s}∼Ga\left(\frac{1}{3},0\right)\text{.}$$

**Fig. 1:**
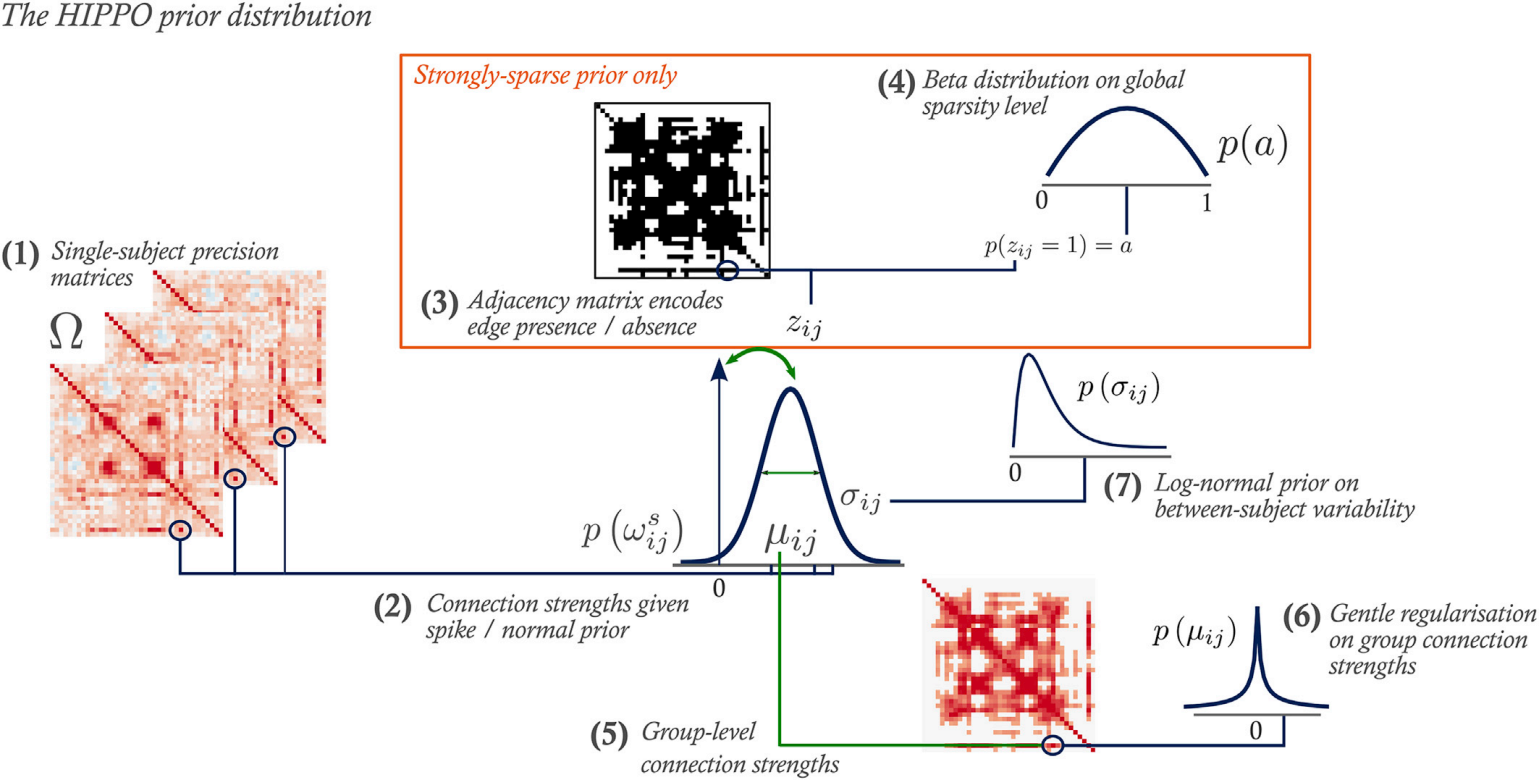
Hierarchical prior on precision matrices. A spike and non-central slab prior *(2)* is placed on each off-diagonal element of the precision matrices *(1)*. In the strongly sparse version of the prior, selection of the slab or spike (presence or absence of a network connection) is controlled by an adjacency matrix *(3)*, with a learnt sparsity level *(4)*. In the weakly sparse model, this feature is not used and only the slab imposed as a prior on connection strengths. The slab is modelled as a normal distribution describing the mean *(5)* and variance over connection strengths. The mean is regularised by a sparsity-inducing prior *(6)*, and the variance by a weakly informative log-normal prior *(7)*. We call this model and its inference scheme Hierarchical Inference of Posterior Precisions in OSL (HIPPO).

The full prior on the off-diagonal elements is given in equation (10), discussed in detail below, and is illustrated in Fig. 1. In essence, it is a spike-and-slab prior with shared sparsity over subjects, normally-distributed connection strengths about the population mean, and regularisation on the mean effect.

(10) $$\begin{array}{lll} \left(\omega_{ij}^{s}|\sigma_{ij},\mu_{ij},z_{ij}=1\right) & ∼ & N\left(\mu_{ij},\sigma_{ij}^{2}\right)\\ \left(\omega_{ij}^{s}|z_{ij}=0\right) & ∼ & \delta_{0}\\ \log\;\sigma_{ij} & ∼ & N\left(\log\;m_{\sigma },s_{\sigma }^{2}\right)\\ \mu_{ij} & ∼ & N\left(0,\chi^{2}\right)\\ \chi & ∼ & C^{+}\left(0,A\right)\\ z_{ij} & ∼ & \text{Bernoulli}\left(a\right)\\ a & ∼ & \text{Beta}\left(a_{\pi },b_{\pi }\right) \end{array}$$

Each off-diagonal element of the precision matrices is given a spike and non-central slab prior. The spike, a delta-function at zero, imposes a common sparsity structure over all subjects, using edge inclusion variables Z. For those edges that are included, the non-central slab is a normal distribution, whose variance $\sigma_{ij}$ characterises the between-subject variability of that particular connection strength, and whose mean $\mu_{ij}$ captures the group-level behaviour.

Following advice in Gelman (2006) and Polson and Scott (2012) on the inference of higher-level group parameters in regression, we apply regularisation to the mean edge strengths (μ) towards zero using a normal distribution, learning the rough scale of these connectivities, $\chi^{2}$, from the data and pooling this information over all the edges. This learnt variance parameter $\chi^{2}$ is constrained with a proper, sparsity-promoting, weakly informative prior. Gelman and Polson & Scott recommend the half-Cauchy distribution for this application, denoted $C^{+}$, because it has a finite mass at zero, is undefined for negative values, drops off near a scaling point *A*, and has a heavy tail which can allow the likelihood to dominate. An additional advantage is that the half-Cauchy and can be expressed in a conditionally-conjugate fashion through a scale mixture of normals (Gelman, 2006; Gelman et al., 2008; Polson and Scott, 2012). This parameter expansion technique ensures that sampling for the group-level parameters requires only simple draws from multivariate normal distributions, and these parameters can be integrated over when sampling the top-level edge inclusion variables Z. An alternative choice to the Cauchy, from the same family but imposing stronger sparsity on the group connection strengths, would be the Laplace or LASSO prior (Carvalho et al., 2010). The scale of the Cauchy distribution can be set sensibly based on the data: we use A=0.7 as an appropriate value for variance-scaled data where correlations and partial correlations do not frequently exceed this number.

A broad normal prior is placed on the logarithm of $\sigma_{ij}$, centred on $m_{\sigma }=0.5$ and with a standard deviation $s_{\sigma }=1$ to allow order-of-magnitude deviations from this value. Finally, a Beta-Bernoulli conjugate prior is placed on the edge inclusion variables, with a shared sparsity parameter *a* inferred from the data. The hyper-parameters in the top-level Beta distribution can be set to weakly encourage levels of sparsity encountered in functional networks. Using values of $\left(a_{\pi },b_{\pi }\right)=6$ places most of the prior mass between 0.3 and 0.7.

The values of the hyper-parameters we use are set out in Table 1.

**Table 1:** Values of hyper-parameters employed for functional network modelling.

Parameter	Value
$m_{\sigma }$	0.5
$s_{\sigma }$	1
*A*	0.7
$a_{\pi }$	6
$b_{\pi }$	6

#### Model 2: a weakly sparse prior

The explicit sparsity can be removed from the model by setting a=1 and $z_{ij}=1\;\forall \;i,j$ to create a *weakly sparse* prior. It is weakly sparse in the sense that the group mean connectivities are still shrunk towards zero, using the Cauchy prior, and the subjects' precisions are distributed about these connection strengths. However, the model for the underlying GGM assumes a full graph, and the connectivity estimates should therefore be less sparse than from the first model. Inference is performed in exactly the same fashion as for the strongly sparse model, but without the need for updates on Z.

### Model inference

The procedure taken for inference on the HIPPO model is described in full in supplementary information A. The sampler moves through a series of Gibbs steps, based on (7) and (8) above, in which all of the variables associated with a single column of the matrices are drawn together, $p\left(\left\{\omega_{12}^{s}\right\},\mu_{12},z_{12}\;|\;\left\{\Omega_{11}^{s}\right\},\sigma ,\chi ,a\right)$. Within each of these column-conditionals, we exploit ideas from Peltola et al. (2012) to collapse the conditional distribution over edge-strength parameters ω and μ. This leaves a simple Metropolis-Hastings (MH) sampler on $z_{12}$ at the top level, checking for network edges to add or remove by testing the model evidence of each proposal. The parameters describing group-average and subject-level connectivities can then be sampled directly.

Draws from the posterior distribution of each subject's precision matrix can be used to construct a posterior over correlation or partial correlation matrices, and on the group average of these quantities. We use the posterior mean of the partial correlation distribution as a summary estimate of each subject's functional connectivity.

## Methods

### Evaluating sparse connectivity estimation using simulated data

#### Data generation

Ten simulated datasets were created to test the hierarchical models over a range of sparse network structures, model sizes, quantities of data, and amount of subject variability in connectivity. Except for some minor differences detailed below, each dataset consists of a number of subjects, with an individual precision matrix to represent each subject's functional connectivity; the data for each subject is a draw of samples from a zero-mean multivariate normal distribution, using the appropriate precision matrix. The general properties of each simulation are summarised in Table 2.

**Table 2:** Description of simulated datasets used in Fig. 2. Datasets are characterised by their size (number of subjects, network nodes and links, and data samples), the amount of subject variability (expressed as the standard deviation of connection strengths, over subjects, divided by the mean connection strength; we take the mean of this coefficient of variation over all connections present in the network), the sparsity of the network, and the type of network structure. We use simple circle models of varying sizes, first set out in Wang (2012b) and used in Wang (2012a) and Hinne et al. (2015); together with the largest simulation (netsim 4) from Smith et al. (2011); a random graph structure; and connection matrices built on estimates of the networks in cat cortex (Scannell et al., 1999) and macaque visual cortex (Felleman and Van Essen, 1991).

ID	Subjects	Nodes	Links (full model)	Links	Samples	Variability	Sparsity	Model Structure
1	5	6	15	6	18	0	0.60	Circle
2	25	6	15	6	18	0	0.60	Circle
3	25	6	15	6	50	0	0.60	Circle
4	25	6	15	6	100	0	0.60	Circle
5	50	50	1225	61	200	0	0.95	Netsim 4
6	25	25	300	216	500	0.2	0.28	Random
7	25	6	15	6	25	0.5	0.60	Circle
8	25	30	435	21	100	1.1	0.93	Circle
9	30	52	1326	438	500	1.8	0.67	Cat cortex
10	25	30	435	161	100	2.7	0.63	Macaque visual cortex

*Circle models* Simulations 1–4, 7 and 8 use a simple circular network structure. This structure has been used extensively in the sparse GGM literature: it was set out in Wang (2012b), and used in Hinne et al. (2015); Mohammadi and Wit (2015); and Wang (2012a). A precision matrix of any dimension *p* is constructed as $\omega_{ii}=1$, $\omega_{i,i+1}=0.5$, and $\omega_{1,p}=0.4$, with the lower diagonal elements matched to ensure symmetry.

Simulations 1–4 use the same precision matrix for each subject. Simulations 7 and 8 treat the circle model as the group mean matrix, assign random signs to the connection strengths, and draw single-subject connection strengths from a normal distribution about the group mean with a standard deviation of 0.05 and 0.15 respectively. Subjects' connectivity matrices were then adjusted to be positive semi-definite (Higham, 1988)^2^ and rescaled to unit variance.

*Netsim* Dataset 5 is the 4th network simulation from Smith et al. (2011), chosen because the 4th simulation was the largest model in that work. Smith et al. generated autocorrelated fMRI-like data from an asymmetric network model. As we are estimating symmetric precision matrices, we make the simple assumption that all subjects share the same precision matrix, and we estimate this ground truth as the unregularised partial correlation computed from the entire concatenated dataset, masked by the symmetrised adjacency matrix of the original simulation.

*Random* Dataset 6 is designed to mimic the prior structure. Each network edge is randomly assigned a probability of 0, 0.5 or 1 of being included in the network. Subjects' connection strengths on each edge are normally distributed about 0.25 with a standard deviation of 0.05. As each data point is drawn for each subject, edges in the model are turned on or off in accordance with their probability of edge inclusion. The ‘true’ matrix for each subject is the product of the edge inclusion probability matrix and the subject's connection strengths.

*Cat cortex and macaque visual cortex* Simulations 9 and 10 use the structures of mammalian cortical networks as their basis. Simulation 9 employs the cat cortical network from Scannell et al. (1999) and simulation 10 that of the macaque visual cortex from Felleman and Van Essen (1991)^.^^3^ In each case, the network connection matrix is binary and asymmetrical. We used only the upper triangular part, symmetrising to the lower half. For each simulation, the group mean connection matrix was drawn from a conditional *G*-Wishart distribution (Lenkoski, 2013) with identity scale matrix and degrees of freedom equal to one less than the number of network nodes. Individual subjects' network matrices were drawn from a conditional *G*-Wishart distribution using the mean connection matrix as the scale, and degrees of freedom set to 50 for the simulations from macaque visual cortex and 400 for those from cat cortex. The *G*-Wishart distributions were constrained using the relevant network matrices (cat, macaque) as the underlying graph.

#### Models tested

Sixteen different models were fitted to the test datasets, representing the range of methods in current use for covariance modelling from the most naïve to the most advanced. Their basic properties are set out in Table 3.

**Table 3:** Characterisation of methods used on simulated data in Fig. 2. We classify the methods under test by their inference method, the style of sparsity imposition, and whether they are fitted to individual subjects, the concatenated dataset, or infer individual connectivity matrices using information from the whole group. HIPPO is our acronym for the hierarchical sparse Bayesian model.

Name	Fitted to	Sparsity	Inference	Reference
Partial correlation	individuals	none	analytic	Fisher (1924)
Tikhonov	individuals	continuous	optimised	
Graphical LASSO (GLASSO)	individuals	continuous	optimised	Mazumder and Hastie (2012b)
Group GLASSO (Varoquaux)	group	continuous	optimised	Varoquaux et al. (2010)
Group GLASSO (Danaher)	group	continuous	optimised	Danaher et al. (2015)
Fused GLASSO	group	continuous	optimised	Danaher et al. (2015)
Sparse Group Gaussian Graphical Model (SGGGM)	group	continuous	optimised	Ng et al. (2013)
Wishart	individuals	continuous	analytic	Gelman et al. (2014)
Hierarchical Wishart	group	none	MCMC	Marrelec et al. (2006)
Bayesian GLASSO	individuals	continuous	MCMC	Wang (2012a)
Stochastic Search Variable Selection (SSVS)	individuals	normal-mixture	MCMC	Wang (2015)
*G*-Wishart	concatenation	spike & slab	MCMC	Hinne et al. (2015)
Bayesian Multiple Gaussian Graphical Models (MGGM)	group	spike & slab	MCMC	Peterson et al. (2015)
Single-subject HIPPO	concatenation	spike & slab	MCMC	
Weakly-sparse HIPPO	group & individuals	continuous	MCMC	
Strongly-sparse HIPPO	group & individuals	spike & slab	MCMC	

*Partial correlation* The sample covariance matrix for each subject is inverted, using the Cholesky algorithm, and normalised to produce the unregularised partial correlation matrix.

*Tikhonov* A Tikhonov-regularised estimate of the precision matrix is constructed by slightly augmenting the diagonal of the sample covariance matrix,

(11) $$\hat{\Omega }={\left(\Sigma +\lambda I\right)}^{-1}\;\text{.}$$

The regularisation parameter *λ* was chosen to minimise the RMS distance between the subjects' matrices and their unregularised group average.

This is the procedure used by the HCP and the UK Biobank imaging project in their estimation of fMRI network matrices.

*GLASSO* The graphical LASSO algorithm of Friedman et al. (2008), with modifications for computational efficiency (Mazumder and Hastie, 2012a; b), solves the optimisation problem

(12) $$\hat{\Omega }=\underset{\Omega }{\text{arg}\;\text{max}}\left(\log\text{det}\Omega -trace\left(\Sigma \Omega \right)-\lambda ||\Omega |{|}_{1}\right)\;\text{,}$$

where $||\cdot |{|}_{1}$ indicates the $L_{1}$ norm (sum of the absolute values of the elements of the matrix). The regularisation parameter *λ* was chosen to minimise the RMS distance between the subjects' matrices and their unregularised group average. GLASSO is a very common method for estimating partial correlation brain networks, and the most successful tested in Smith et al. (2011), making it a good benchmark for our work.

*Group GLASSO* The group graphical LASSO of Varoquaux et al. (2010) is fitted to all subjects at once, and encourages a similar sparsity pattern across them. It solves the optimisation problem,^4^

$$\left\{{\hat{\Omega }}^{s}\right\}=\underset{\left\{\Omega \right\}}{\text{arg}\;\text{max}}\left(\sum_{s}\left[n_{s}\;\log\text{det}\Omega^{s}-trace\left(\Sigma^{s}\Omega^{s}\right)\right]-\lambda \sum_{i\neq j}{\left(\sum_{s}\omega_{ij}^{s2}\right)}^{\frac{1}{2}}\right)\;\text{.}$$

The regularisation parameters were chosen to maximise the predictive log-likelihood under the default cross-validation scheme, which repeatedly narrows down the hyper-parameter search space.

The group graphical LASSO of Danaher et al. (2015) is a generalisation of Varoquaux et al.'s model. It solves the optimisation problem,

(13) $$\left\{{\hat{\Omega }}^{s}\right\}=\underset{\left\{\Omega \right\}}{\text{arg}\;\text{max}}(\sum_{s}[n_{s}\;\log\text{det}\Omega^{s}-trace\left(\Sigma^{s}\Omega^{s}\right)]-\lambda_{1}\sum_{s}\sum_{i\neq j}|\omega_{ij}^{s}|-\lambda_{2}\sum_{i\neq j}{\left(\sum_{s}\omega_{ij}^{s2}\right)}^{\frac{1}{2}})$$

The regularisation parameters were chosen to minimise the Bayesian information criterion associated with this likelihood. Inference was performed in Matlab.

*Fused GLASSO* The fused graphical LASSO of Danaher et al. (2015) is also fitted to all subjects at once, and seeks to impose collective sparsity on all subjects' network elements, while encouraging networks from different subjects to be alike. Inference is set up as an optimisation problem with two penalty terms, solved using alternating directions method of multipliers (ADMM),^5^

(14) $$\left\{{\hat{\Omega }}^{s}\right\}=\underset{\left\{\Omega \right\}}{\text{arg}\;\text{max}}\left(\sum_{s}\left[n_{s}\;\log\text{det}\Omega^{s}-trace\left(\Sigma^{s}\Omega^{s}\right)\right]-\lambda_{1}\sum_{s}\sum_{i\neq j}|\omega_{ij}^{s}|+\lambda_{2}\sum_{s}\sum_{s'>s}\sum_{i,j}|\omega_{ij}^{s}-\omega_{ij}^{s'}|\right)$$

The regularisation parameters were chosen to minimise the difference between the group mean connectivity inferred using half of the dataset and the unregularised mean of the other half of the dataset.

*SGGGM* The Sparse Group Gaussian Graphical Model (SGGM) proposed by Ng et al. defines group-level connection strengths, and regularises each subject's estimates towards this central representation. A restricted maximum-likelihood solution, found using ADMM,^6^ solves the optimisation problem,

(15) $$\left\{{\hat{\Omega }}^{s}\right\}=\underset{\left\{\Omega \right\}}{\text{arg}\;\text{max}}\left(\sum_{s}\left[n_{s}\;\log\text{det}\Omega^{s}-trace\left(\Sigma^{s}\Omega^{s}\right)\right]-\lambda_{1}\sum_{i\neq j}|\omega_{ij}^{G}|+\lambda_{2}\sum_{s}{\left(\sum_{i,j}|\omega_{ij}^{s}-\omega_{ij}^{G}{|}^{2}\right)}^{2}\right)$$

By imposing sparsity on the group network, and using a Frobenius norm penalty on the difference between elements of subjects' matrices and the group, it has a hierarchical structure that is very similar in form to the weakly sparse Bayesian hierarchical model that we propose. The regularisation parameters were chosen to minimise the distance between the group mean connectivity inferred using half of the dataset and the unregularised mean of the other half of the dataset.

*Wishart* Following Gelman et al. (2014), a simple Wishart prior distribution is placed independently over each subject's precision matrix, $\Omega ∼W_{p}\left(p+1,\;\frac{1}{p+1}I\right)$. This leads analytically to the posterior for each subject, which we summarise by its expectation.^7^ The similarity to Tikhonov regularisation is clear,

(16) $$p\left(\Omega^{s}|S\right)=W_{p}\left(\Omega^{s};\;p+1+n_{s},{\left(\left(p+1\right)I+S\right)}^{-1}\right)\text{.}$$

*Hierarchical Wishart* Marrelec et al. (2006) proposed a hierarchical model for the covariance structure of fMRI recordings. No encouragement of sparsity was introduced in the prior structure, but it makes a useful comparison point for our hierarchical models. Rather than Marrelec et al.'s hierarchy of Inverse-Wishart distributions on covariance matrices, we use an equivalent hierarchy of Wishart distributions on precision matrices,

(17) $$\Omega^{s}|B∼\;W_{p}\left(\nu_{0},B^{-1}\right)\;\text{.}$$

Marrelec et al. do not mention placing a hyperprior on the group-level parameters, so we presume they used a flat prior, B∼1. We prefer to use a very weakly informative prior, and follow Hinne et al. and Gelman et al. in selecting a very weak Wishart hyperprior for the group connection strengths,

(18) $$B∼\;W_{p}\left(3,I\right)\;\text{,}$$

where I is the identity matrix. This model leads to a simple Gibbs inference scheme,

(19) $$p\left(\Omega^{s}|S,B\right)=\;W_{p}\left(\Omega^{s};\;\nu_{0}+n_{s},{\left(B+S\right)}^{-1}\right)$$

(20) $$p\left(B|\Omega^{s}\right)=\;W_{p}\left(B;\;N\nu_{0}+3,{\left(I+\sum_{s}\Omega^{s}\right)}^{-1}\right)\;\text{.}$$

Marrelec et al. also did not discuss methods for inferring the degrees of freedom of the group-level prior, $\nu_{0}$, which controls the strength of the regularisation. There is no simple conjugate hyperprior that can be used, so we take a simple empirical approach. We set $\nu_{0}$ to be the value that, under 5 bootstrapped cross-validation splits of the subjects into two halves, minimises the error between the mean of the partial correlation matrices inferred with the hierarchical Wishart model and the mean of the remaining partial correlation matrices inferred with the GLASSO, using mild regularisation (*λ* = 0.01). We run the Gibbs sampler for 1500 iterations, using an additional 1000 as warm-up.

*Bayesian GLASSO* The Bayesian graphical LASSO of Wang (2012a) places a Laplace or double-exponential prior on the off-diagonal elements of the precision matrix,

(21) $$\pi \left(\omega_{ij}\right)=\frac{\lambda }{2}\text{exp}\left(-\lambda |\omega_{ij}|\right)\;\text{.}$$

For each subject, 3000 samples were drawn using Wang's algorithm,^8^ after discarding 1000 as warm-up.

*SSVS* The Bayesian Stochastic Search Variable Selection algorithm (Wang, 2015) for covariance selection places a mixture of normal priors on the off-diagonal elements of the precision matrix,

(22) $$\pi \left(\omega_{ij}\right)=\left(1-a\right)N\left(\omega_{ij};\;0,v_{0}^{2}\right)+a\;N\left(\omega_{ij};\;0,v_{1}^{2}\right)\;\text{,}$$

where $v_{0}$ is chosen to be much smaller than $v_{1}$ and sampling proceeds using Gibbs sampling along the columns. We follow Wang's recommendations and set $v_{0}$ to 0.05, $v_{1}$ to 2.5 and *a* to 0.5. For each subject, 3000 samples were drawn after 1000 warm-up samples.^9^ Neither this model, nor the Bayesian GLASSO above, has been used for neuroimaging, to our knowledge.

*G-Wishart* The *G*-Wishart distribution is the conjugate prior on the multivariate normal, describing a single precision matrix Ω, conditional on the graph G on which it is supported,

(23) $$\begin{array}{lll} \pi \left(\Omega |G,\delta ,V\right) & = & W_{G}\left(\delta ,V\right)\\ & = & \frac{{\left|\Omega \right|}^{\frac{\delta -p-1}{2}}}{Z_{G}\left(\delta ,V\right)}\exp\left[-\frac{1}{2}trace\left(V^{-1}\Omega \right)\right]1_{\Omega \in {\mathbb{P}}_{G}}, \end{array}$$

where V is the scale matrix, *δ* indicates the degrees of freedom, $Z_{G}$ is the intractable normalising constant, and Ω is constrained to live on the cone ${\mathbb{P}}_{G}$ of positive definite p×p matrices with zeros indicated by the graph G.

Most Bayesian sparse precision modelling efforts have focussed on this prior. Sampling from the *G*-Wishart distribution, conditional on a known graph G, can be performed easily (Lenkoski, 2013). Sampling from the joint distribution (Ω,G) is much harder. A scalable inference solution is still elusive, and no attempt has been made at a hierarchical model that could learn the scale matrix V. The most efficient *G*-Wishart approach perhaps is set out in Hinne et al. (2015), which applies the model for inference of subcortical functional connectivity in fMRI.

We fitted the *G*-Wishart model to the entire dataset concatenated over subjects, using software provided by Hinne et al.,^10^ using 5000 warm-up samples and 10 000 draws from the distribution. We follow Hinne et al. in using an uninformative prior specification, δ=3 and $V=I_{p}$. Fitting the model to the concatenated data provides a useful comparison to the hierarchical models, using all of the data for inference, but assuming that each subject shares the same network matrix. It would not be computationally feasible to run the *G*-Wishart algorithm separately on individual subjects for the larger network models.

*MGGM* The Bayesian Multiple Gaussian Graphical Models (MGGM) approach, proposed by Peterson et al., is a hierarchical generalisation of the network structure used by the *G*-Wishart model. It posits that each subject (or sub-group) can have a different graphical model structure (although it shares no information about the connection strengths), and links these models using a Markov random field (MRF) prior,

(24) $$\begin{array}{l} \pi \left(\Omega^{s}|G_{s},\delta ,V\right)=W_{G_{s}}\left(3,I\right)\prod_{i<j}p\left(g_{ij}|v_{ij},\Theta \right)\\ p\left(g_{ij}|v_{ij},\Theta \right)\propto \;\exp\left(v_{ij}1^{T}G_{ij}+g_{ij}^{T}\Theta g_{ij}\right) \end{array}$$

where the 1×S binary vector $g_{ij}$ defines the presence of an edge in each subject, the edge-specific hyper-parameter $v_{ij}$ indicates the likelihood of an edge and is given a Beta hyper-prior. The S×S symmetric matrix Θ encodes the pairwise similarity of each graph $G_{s}$, and is in turn given a spike-and-slab prior.

Unfortunately, the flexibility of this MRF prior also brings complexity: the computational burden of Peterson et al.'s algorithm^11^ scales as $2^{S}$. Allowing each subject to have its own sparse model structure becomes infeasible for most practical purposes. We tested the performance of the model only for our first simulated dataset of five subjects, using 5000 warm-up samples and 10 000 draws from the posterior. The model would be more practical for exploring differences in network structure between two or three groups of subjects.

*Single-subject HIPPO* A model based on the sparse hierarchical prior presented here (equation (10)), but simplified for single-subject inference, was designed as a comparison to the performance of the *G*-Wishart model. The prior can be expressed as

(25) $$\begin{array}{l} \left(\omega_{ii}|\lambda \right)∼\;Exp\left(\frac{\lambda }{2}\right)\\ \left(\omega_{ij}|z_{ij}=1\right)∼\;N\left(0,0.7^{2}\right)\\ \left(\omega_{ij}|z_{ij}=0\right)∼\;\delta_{0}\\ z_{ij}∼\;\text{Bernoulli}\left(a\right)\\ a∼\;\text{Beta}\left(6,6\right)\\ \lambda \;∼\;Ga\left(\frac{1}{3},0\right)\;\text{.} \end{array}$$

Inference follows the format above. We use a single chain for inference, drawing 5000 warm-up samples and 10 000 samples from the distribution.

*Weakly-sparse HIPPO* The HIPPO hierarchical model set up without the explicit sparsity prior. There is still regularisation of the group connection strengths towards zero—in this sense, it is *weakly sparse*. Inference is the same as under the strongly sparse HIPPO model, but conditional on a full graph: all edge inclusion variables $z_{ij}$ are set to 1 (as described in section 2.3.2, Cauchy priors are chosen that suppress the mean connectivities towards zero with the subjects distributed around this point, without imposing absolute edge sparsity). We draw 30 000 samples, with 10 000 as warm-up.

*Strongly-sparse HIPPO* The full sparse hierarchical model (Hierarchical Inference of Posterior Precisions in OSL) set out in equation (10). We draw 30 000 samples in a single chain, with an additional 10 000 as warm-up.

#### Analysis

After fitting each model, we compute the root-mean-square (RMS) error (over edges) between each subject's simulated connection strengths and the inferred partial correlation matrices. We compare the mean and standard deviation of this metric over subjects. We also compute the area under the receiver-operator characteristic (ROC) curve, which traces the trade-off between specificity and sensitivity in detection of network edges in the simulated sparse network as a threshold is applied to the inferred connection strengths. For all of the Bayesian models, we use the mean of the posterior over partial correlation matrices as the summary estimate of connectivity in each subject.

### Performance evaluation using resting-state data

To evaluate models' ability to accurately reconstruct functional networks using real data, we test how well they can estimate connectivity from very limited samples of fMRI and MEG data. Using the best-performing models, we illustrate two additional analyses. We look at the models' ability to predict subjects' biological and behavioural traits from their fMRI connectomes, and estimate the proportion of variation in MEG functional connectivity that could be attributable to genetic factors.

#### Dataset

We use fMRI data from the first 200 subjects of the Human Connectome Project's HCP900 data release (Van Essen et al., 2013). All subjects provided four 15-min resting-state fMRI scans. We also use the 61 subjects from the MEG2 data release (Larson-Prior et al., 2013), who provided three resting-state MEG scans of 6 mins' duration. All subjects are young (22–35 years of age) and healthy.

A heritability analysis on the MEG data exploits the family structures of the subjects. Of the 61, 28 are monozygotic twins and 16 are dizygotic twins. Zygosity of twin subjects was determined by genotype where available, and otherwise by self report.

HCP data were acquired using protocols approved by the Washington University institutional review board. Informed consent was obtained from subjects. Anonymised data are publicly available online from ConnectomeDB.^12^

#### fMRI preprocessing and predictive analyses

Resting-state fMRI data were acquired with 2 mm isotropic spatial resolution and a temporal resolution of 0.72 s. The HCP provides comprehensively pre-processed data (Glasser et al., 2013) that are registered to a standard cortical surface with the MSMAll algorithm (Glasser et al., 2016; Robinson et al., 2014; a high-quality registration approach that combines descriptions of brain structure, function and connectivity from multiple imaging modalities to precisely align functional regions), and for which structured artefacts have been removed by a combination of independent component analysis (ICA) and FIX (Salimi-Khorshidi et al., 2014), FSL's automated noise component classifier.

We modelled connectivity between the 25 non-contiguous spatial components, computed by group ICA, that are released by the HCP. For simplicity, we fitted our models to the concatenated data over all four scans. We fitted both the strongly sparse and weakly sparse hierarchical models, running three sampling chains for 40 000 samples in the sparse model, with 20 000 needed for convergence in the weakly sparse model, using an additional 10 000 samples as warm-up. Additionally, we fitted Ng et al.'s SGGM, choosing the regularisation parameters to minimise the root mean square distance between individual subjects' partial correlation matrices inferred from half of the available data, and unregularised estimates from the remaining half. Finally, we estimated Tikhonov-regularised precision matrices for each subject. We followed the procedure used for the connectomes released from the HCP, applying only gentle regularisation with *λ* set to 0.01.

Having computed precision matrices for each subject with these three methods, and converted into partial correlations (taking the posterior mean from the Bayesian models as a summary estimate), we fitted linear predictive models to two traits recorded as part of the HCP: sex, and the number of correct scores on a picture vocabulary test. We used the partial correlations on each network edge, for each subject, as the predictors, after regressing out the confounding effects of age, the square of age, sex and an age–sex interaction term,^13^ the cube root of cortical volume and of intra-cranial volume, both computed with Freesurfer, the software version for image reconstruction, and an estimate of each subject's motion in the scanner (rfmri_motion). Sex was predicted using logistic regression with elastic net regularisation (Friedman et al., 2010; Zou and Hastie, 2005).^14^ Scores on the picture vocabulary test were demeaned and standardised, and predicted with linear regression using elastic net regularisation. Parameters for the elastic net were tuned in both cases by two nested loops of 5-fold cross-validation. Performance of the models was assessed by computing accuracy (sex) or correlation between predicted scores and real performance (picture vocabulary task), using a 5-fold cross-validation loop for training and prediction. The stratification of subjects into the cross-validation folds was designed such that families were not split over the fold groupings (Winkler et al., 2015).

#### MEG preprocessing and genetic analyses

Resting-state MEG data were acquired on a whole-head Magnes 3600 scanner (4D Neuroimaging, San Diego, CA, USA). The data were pre-processed to compensate for head movement, to remove artefactual segments of time from the recordings, identify recording channels which are faulty, and to regress out artefacts with clear artefactual temporal signatures (such as eye-blinks or cardiac interference) using ICA (Larson-Prior et al., 2013). Sensor-space data were down-sampled from 509 Hz to 300 Hz, with the application of an anti-aliasing filter.

MEG data from each session were source-reconstructed using a scalar beamformer (Robinson and Vrba, 1999; Van Veen et al., 1997; Woolrich et al., 2011). Pre-computed single-shell source models are provided by the HCP at multiple resolutions, registered into the standard co-ordinate space of the Montreal Neuroimaging Institute. Data were filtered into the 1–30 Hz band before being beamformed onto a 6 mm grid using normalised lead fields. Covariance estimation was regularised using principal component analysis (PCA) rank reduction (Woolrich et al., 2011). The rank was conservatively reduced by five more than the number of ICA components removed during preprocessing. Source estimates were normalised by the power of the projected sensor noise. Source-space data were filtered into the beta (13–30 Hz) band, which is associated with a range of resting-state network profiles (Baker et al., 2014; Brookes et al., 2012, 2011; Colclough et al., 2015; Hipp et al., 2012; Mantini et al., 2007; de Pasquale et al., 2012, 2015) and exhibits strong heritability in its functional connectivity profile (Colclough et al., 2017). We employed the parcellation from Colclough et al. (2016, 2017), which consists of contiguous regions extracted from components of an ICA decomposition of the resting-state fMRI recordings of the first 200 HCP subjects. A single time course was constructed to represent each node, following Colclough et al. (2015), as the first principal component of the ROI, after weighting the PCA over voxels by the strength of the ICA spatial map. This analysis yielded 39 time courses for each resting-state session. Spatial leakage confounds were reduced using a symmetric orthogonalisation procedure (Colclough et al., 2015) to reduce shared signal at zero lag between the network nodes. Lastly, power envelopes of the leakage-reduced ROI time courses were computed by taking the absolute value of the Hilbert transform of the signals, low-pass filtering with a cut-off of 1 Hz, and down-sampling to 2 Hz (Luckhoo et al., 2012). Time courses were concatenated over sessions for the purpose of functional connectivity estimation.

We estimated functional connectivity in the same manner as for the fMRI data, using both the strong and weakly sparse HIPPO models, Ng et al.'s SGGM and lightly Tikhonov-regularised inversion of the sample covariance matrices. Identical inference procedures were followed to the fMRI data.

The mean heritability of functional connectivity was estimated from an ACE model, computed using APACE (Chen et al., 2014).^15^ The ACE model is a linear variance-components decomposition that ascribes a portion of the variability in each phenotype (functional network connection) to either additive genetics (A, $h^{2}$), developmental and common environmental factors shared between twins (C, $c^{2}$) and other unmodelled variabilities and noise sources (E, $e^{2}$). The twin structure of the HCP dataset is sufficient to infer on all three components of the model; see Chen et al. (2014) and Chen (2014) for details. This analysis of heritability replicates previous work (Colclough et al., 2017), although with a smaller set of subjects. To provide comparable results, we followed similar analysis steps by fitting the ACE models to correlation matrices, rather than to partial correlation matrices. (For the Bayesian models, we summarise the connectivity for each subject as the posterior mean of the distribution over correlation matrices.) This decision was originally made as correlation matrices are among the most repeatable forms of network analysis in MEG, with better reliability than partial correlation matrices (Colclough et al., 2016). Heritability ($h^{2}$) was computed for each network edge, after regressing out the effect of age, the square of age, sex, an age and sex interaction, the interaction between sex and the square of age, the cube root of intra-cranial volume and of cortical volume (both estimated with FreeSurfer), a measure of subject motion from fMRI recordings (a proxy as no motion measure is available for the MEG scans), an estimate of the noise passed by the beamformer for each subject, and finally two measures of node power, one formed from the standard deviation of the MEG power envelope and the other from the coefficient of variation of the power envelopes. The mean heritability was computed over the network connections, with 95% bootstrapped confidence intervals estimated using 10 000 sub-samples of the data, and permutation-based *p*-values computed using 1000 relabellings of the twin pairs.

#### Performance evaluations using limited data

Lastly, we estimated functional connectivities with the strongly and weakly sparse hierarchical models (HIPPO) for each meg and fMRI subject using only a small portion of the available data: the first resting-state session (of 6 min) in MEG and the first 5 min of recording for fMRI. This allows us to compare network estimations from limited amounts of data to the assumedly much more accurate estimates derived from the entire dataset. Tikhonov estimates from the full datasets (18 min and 60 min, respectively) were computed using the HCP's standard setting of the regularisation parameter *λ* to 0.01. Additionally on the restricted data samples, we tested network estimation using the same Tikhonov regularisation approach; naïve covariance inversion; the original GLASSO, Varoquaux et al.'s group GLSSO, Ng et al.'s SGGM, Danaher et al.'s fused glasso, and Peterson et al.'s MGGM. Where appropriate, regularisation parameters were chosen to minimise the distance between individual subjects' network matrices inferred from half of the available data, and unregularised estimates from the remaining half.

## Results

### Inference of simulated sparse networks

**Fig. 2:**
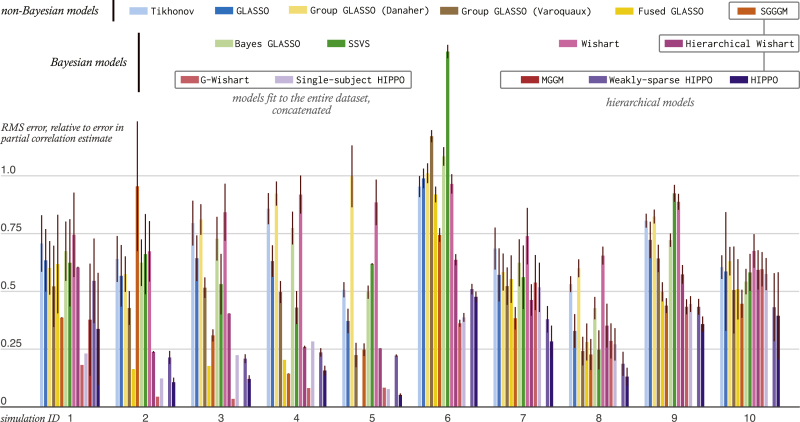
Comparison of sparse network modelling methods on simulated datasets. The rms error between the simulated precision matrices and estimated precision matrices is shown for 10 multi-subject artificial datasets, using 15 different models for inference (indexed by colour). The error is expressed as a proportion of the rms error from a simple partial correlation estimate (naïve matrix inversion). Bars indicate the mean error over subjects, with the standard deviation over subjects given by the associated dark red line. Datasets 1–5 have no subject-variability in the simulated networks; subject variability increases through datasets 6–10. With limited subject variability, models fitted to the concatenated data perform the best. As this variation increases, the Bayesian hierarchical models win out. There is no result for the fused GLASSO on simulation 5, because the model would not run in a feasible time-frame (it would take longer than a week). Inference for the MGGM approach was only possible in the first, six-subject dataset.

The performance of the strongly sparse and weakly sparse Bayesian hierarchical models presented in section 2 is compared with that of 13 additional models, over 10 different simulated datasets, in Fig. 2. The models are summarised in Table 3 and the datasets in Table 2. It is worth noting some general trends. Firstly, as the amount of data increases for inference, covariance estimation becomes less noisy and the error in reconstruction goes down (Fig. 2, simulations 1–4). Of particular note is the difference between datasets 1 and 2, where the number of subjects increases but the amount of data per subject is constant. The hierarchical Bayesian models are able to use this increase in information to reduce reconstruction error, whereas models fitted to individual subjects self-evidently are not. Secondly, attempting to perform inference on precision matrices without any form of regularisation is in general a bad idea: all methods tested outperform the simple inversion of the sample covariance matrix. Thirdly, in these simulations of sparse networks, models which build in explicit sparsity with spike-and-slab priors (the *G*-Wishart models and the single- and multi-subject sparse HIPPO models) show improved reconstruction compared to models with differentiable regularisation terms (e.g. GLASSO) or continuous priors (the Bayesian GLASSO and the weakly sparse HIPPO). Fourthly, in simulations with little-to-no between-subject variability, models fitted to the concatenated group data perform the best (unsurprisingly). However, as the variability between subjects increases, the hierarchical models that allow individual subject network estimation win out.

The best performing non-Bayesian model is Ng et al.'s hierarchically-structured SGGM. In a similar fashion to our weakly sparse HIPPO prior, it uses a regularisation term to encourage similarity between subjects' networks and the group connectivity, as well as suppression of group-level connectivities towards zero. It performs well on the small datasets with no subject variability, although the strongly sparse Bayesian hierarchical model produces better estimates on datasets 1–3 (albeit by a very small margin). On datasets 5–10, SGGM is beaten by both hierarchical Bayesian models.

**Fig. 3:**
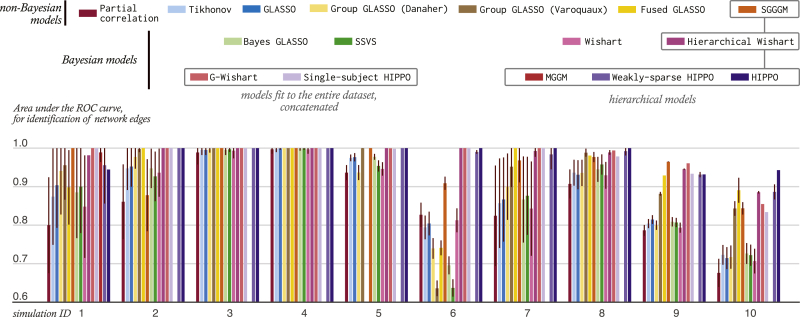
Comparison of network modelling methods' ability to discover network structure. The area under the ROC curve for discovery of the underlying graph structure is shown for 16 models (indexed by colour) applied to 10 multi-subject artificial datasets. Bars indicate the mean area under the curve over subjects; the standard deviation over subjects is given by the dark red line. All subjects in these datasets share the same network structure, so the models fit to the concatenated data perform well. Apart from these, the sparse hierarchcal models (both Bayesian and not) generally outperform the rest. A score of 1.0 for a particular model indicates that there exists a threshold that perfectly identifies the network graph when applied to the inferred connections. A score of 0.5 indicates no better performance than chance.

We also evaluated each model's ability to discover the underlying structure of the simulated GGMs. Fig. 3 shows the area under the ROC curve for each model, indicating its ability to identify the GGM of each dataset. In general, Bayesian models with explicit sparse priors (the *G*-Wishart and the sparse HIPPO models) outperform Bayesian models with continuous priors. Ng et al.'s SGGM is the best-performing non-Bayesian model. It underperforms relative to the HIPPO models on datasets 6, 8 and 10, but outperforms them on datasets 1 and 9, giving no clear overall best performer.

No results are given for the fused GLASSO on the largest dataset, number 5, or for the MGGM approach on datasets other than the first, because inference times exceeded a week, without convergence.

In summary, these simulations demonstrate that more accurate individual-subject connectivity estimation is possible with our hierarchical Bayesian framework than with the existing approaches. Discovery of graphical model network structure, too, is at least as good as the state of the art. Close competition comes from the SGGM approach, which, although not Bayesian, has a very similar hierarchical model structure.

### Network estimation from limited datasets

**Fig. 4:**
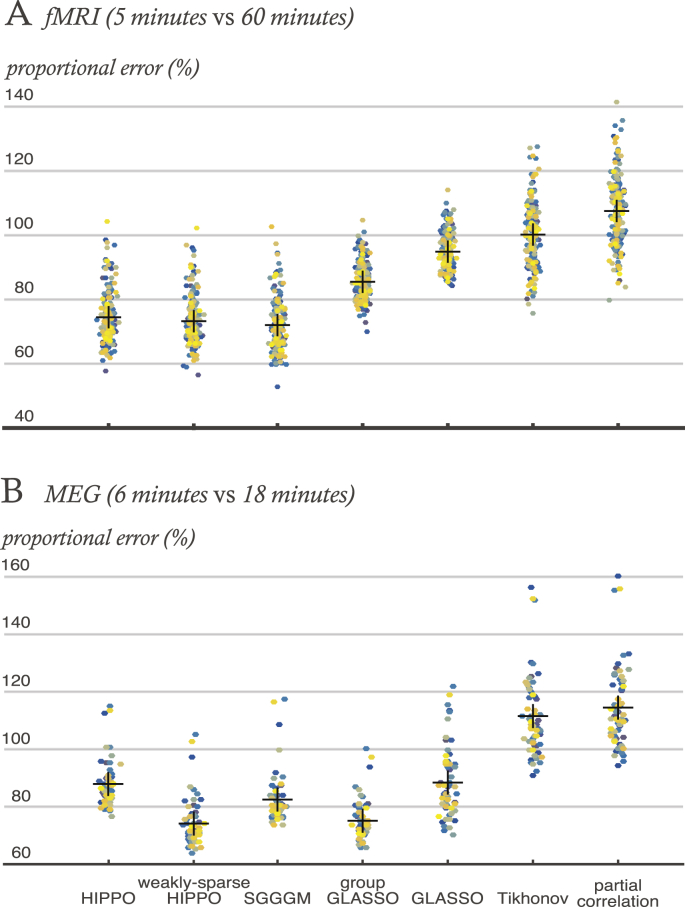
Estimation of network matrices from small samples of data. Single-subject networks estimated from the first 5 min of resting-state fMRI data (A), and single-subject networks estimated from the first resting-state session of MEG recordings in the beta band (B), were compared to the average of each subject's complete data, for each modality. Networks were estimated using the strongly and weakly sparse hierarchical model HIPPO, Ng et al.'s SGGM, with the graphical LASSO, Varoquaux et al.'s group GLASSO, with Tikhonov regularisation and with unregularised partial correlation. The results were compared in each subject to a mildly Tikhonov-regularised estimate from all three sessions' concatenated data; the RMS error from this estimate is displayed as a percentage of the mean connectivity of each subject's network matrix. Coloured dots identify individual subjects. Black crosses denote the mean of each distribution.

A useful metric for assessing improvements to network estimation using real data is the ability of a model to estimate connectivity from a short section of a recording. We compare beta-band network matrices inferred from resting-state MEG recordings from the HCP, using either all three sessions of data, or only a single 6-min session of data. Additionally, we compare fMRI network matrices inferred from resting-state HCP data, using either all four sessions of 15 min, or only the first 5 min of recording. Treating networks inferred from the full datasets using mild Tikhonov regularisation (*λ* = 0.01) as a good approximation of ‘the truth,’ Fig. 4 compares the RMS differences to these estimates from seven inference methods, which only had access to the first portion of the data.

In the fMRI data, the two Bayesian hierarchical models (HIPPO) and the hierarchically-structured SGGM significantly outperform the standard regularised solutions, producing a reduction in error that is on the same order of magnitude as the subject-to-subject variation in this metric. They also outperform the group GLASSO of Varoquaux et al. (2010), which performed well on our simulated data. In the MEG data, the weakly sparse HIPPO model performs similarly well, while the strongly sparse Bayesian model and SGGM are not able to beat the group GLASSO.

A paired *t*-test for a difference in mean performance between the hierarchical models (including SGGM) and the group GLASSO, conducted non-parametrically using 5000 sign flips of the difference between pairs, gave p<0.001 for each hierarchical model in the fMRI data, and p<0.01 for the weakly sparse hierarchical model in the MEG data, without adjusting for the multiple tests. These results equate to a mean improvement in estimation compared to GLASSO, with standard deviation over subjects, of 18±6% (0.7±7%) for the sparse HIPPO model, 19±5% (14±6%) for the weakly sparse HIPPO model, 20±4% (6±7%) for sggm and 8±2% (14±6%) for the group GLASSO on the fMRI (MEG) data.

It is worth noting that the weakly sparse hierarchical model can beat the original GLASSO, in both modalities, even when the regularisation parameter for the latter is chosen *with knowledge of the correct solution*—in other words, when it is allowed to cheat. If *λ* is chosen for each subject so as to minimise the difference between the estimated network and the solution used here as truth, the proportional error with standard deviation over subjects for the GLASSO estimate is 82±8% for fMRI and 83±7% for MEG. The hierarchical Bayesian model can therefore reduce network estimation error to an extent that is better than GLASSO would ever be able to achieve.

The fused graphical LASSO of Danaher et al. and Peterson et al.'s MGGM approach, other models that performed well on simulations, did not approach convergence on these short datasets even after five days of computation on a MacBook Pro (with a 2.8 GHz processor and 16 GB of RAM). Cross-validation of the parameters and computation of a solution was therefore unachievable in a sensible time frame, and results from these methods are not available for comparison.

### Heritability of MEG functional connectivity

To further illustrate the performance of the hierarchical models, we repeated a previous analysis of the heritability of functional connectivity with HCP data (Colclough et al., 2017) using the best-performing models from our evaluations: the two Bayesian hierarchical models and sggm. The hcp dataset is a twin study, and the variability within the functional connectomes of the subjects is determined, in part, by genetic and shared environmental effects (Colclough et al., 2017). Heritability, $h^{2}$ (A), is the proportion of variance in a phenotype that can be explained by additive genetic factors. It is estimated using linear decompositions of the variance into heritability, the environmental effect shared between twins, $c^{2}$ (C), and any other unmodelled variance sources and noise, $e^{2}$ (E). Improving the quality of network matrix estimation, therefore, with a hierarchical model that is blind to the twin structure of the data, should reduce estimates of $e^{2}$ and increase estimates of heritability.

**Fig. 5:**
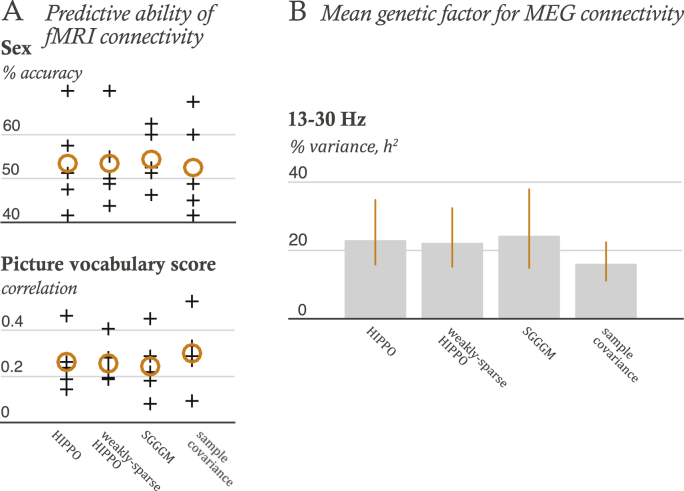
Performance of the hierarchical model. (A) *Prediction of sex (top) and picture vocabulary scores (bottom) from fMRI functional connectomes.* The correlation between scores on a picture vocabulary test and the predicted scores after training a regression model using resting-state fMRI functional connection strengths is shown, together with the accuracy in prediction of subjects' sex using a similar logistic regression model. Results are presented for standard Tikhonov-regularised network inference, and for networks inferred using the weakly sparse and strongly sparse versions of the hierarchical model (HIPPO), and for the hierarchically-structured SGGM. Black crosses indicate results from each of five cross-validation folds; the orange circle indicates the mean of these scores. (B) *Estimation of the mean heritability of functional connections in the MEG beta band.* Heritability estimates are compared between the two versions of the Bayesian hierarchical model, SGGM, and estimation using sample covariance matrices, as performed for Colclough et al. (2017). Bars give the estimated proportion of variability attributed to additive genetics, on average over all connections, and the error lines denote 95% bootstrapped confidence intervals.

We fitted ACE models on each edge, and analysed the average heritability over the edges, computing bootstrapped confidence intervals and permutation-based tests for significance (results shown in Fig. 5B). To allow easy comparison to the previous work, we fitted the models to correlation matrices: those estimated from the sample covariance matrix, from the SGGM model, and correlation matrices estimated from inversion of precision matrices, regularised using the hierarchical inference procedure. Using the hierarchical models, the estimates of heritability (with 95% confidence intervals in square brackets) increased from 16% [11%, 22%] (original estimate) to 22% [15%, 32%] (weakly sparse HIPPO), 23% [16%, 35%] (sparse HIPPO) and 24% [15%, 38%] (SGGM). (Uncorrected permutation-based *p*-values computed for each respective model are 0.01, 0.01, 0.003 and 0.02.) This increase in heritability is related to a reduction in the residual variance (noise and any other factors unexplained by genetics, shared environmental, maternal effects, motion, age, sex or brain size) of 7 percentage points for the Bayesian models and 10 percentage points for SGGM, from an original estimate of 76% for $e^{2}$. This corresponds to a noise reduction of about 10% by using these hierarchical models. These differences are not explained by any random variation in noise or sampling, as exactly the same data were passed into each of the covariance models. Group mean networks for the four models are given in the supplementary information.

### Trait prediction using fMRI functional connectivity

The HCP has released an analysis of the ability of functional connections to predict a wide range of biological and behavioural traits,^16^ using all 841 subjects with resting-state fMRI recordings, suggesting that in some cases there is discriminative information embedded within the functional connectome. To illustrate the application of hierarchical models to predictive modelling, we target two of the megatrawl's more successful traits, scores on a picture vocabulary test (a measure of crystallised intelligence^17^), and sex. Fig. 5A presents a comparison of predictive performance on these two measures using partial correlation networks estimated using Tikhonov regularisation (the algorithm employed in the HCP's disseminated networks), SGGM, and the (posterior mean) partial correlation networks inferred using the strongly sparse and weakly sparse hierarchical models. In all cases, the differences in predictive ability between the models is smaller than the error on the cross-validated estimate (although there is no difference in sampling or random variation between the methods). The correlation between scores on the picture vocabulary test and the predicted responses are slightly worse (by a few percent) for the hierarchical models, whereas there is a slight improvement in accuracy for the prediction of biological sex (although the accuracies are so close to 0.5 that it is difficult to have confidence in the performance of any model). We were principally interested in the differential prediction ability of the models, so permutation tests to look for significant classification have not been performed.

**Fig. 6:**
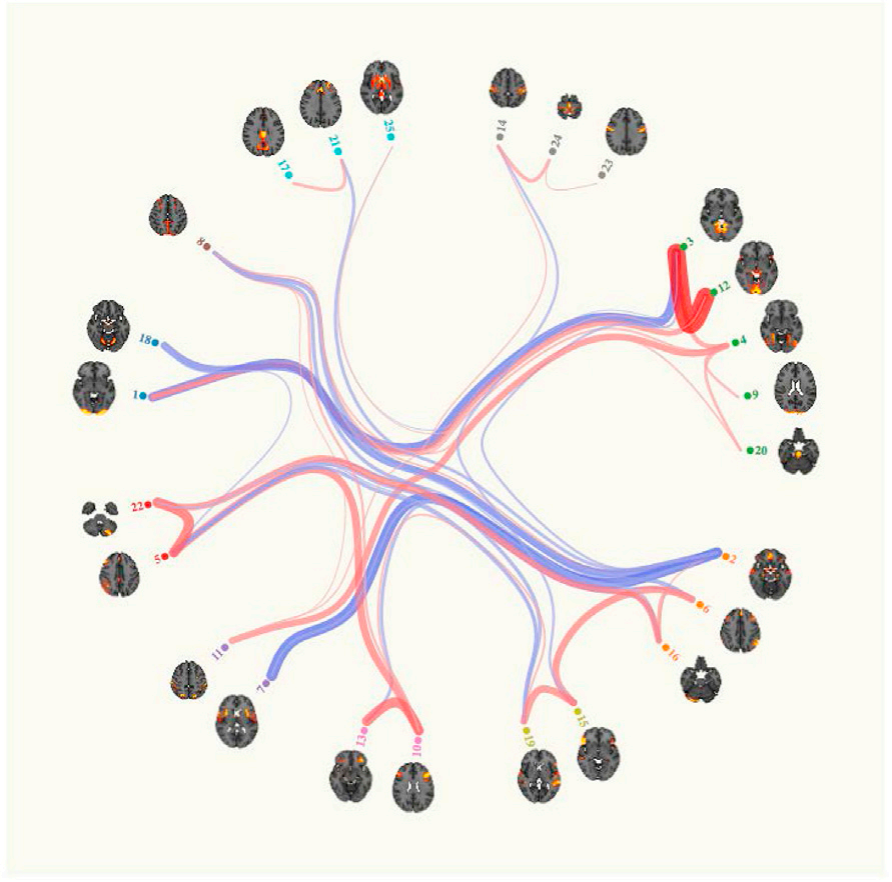
Group average functional network for the HCP fMRI data. Posterior mean of the group average partial correlation network, computed using the hierarchical model (HIPPO). The results obtained using the Tikhonov-regularised, SGGM, or weakly sparse HIPPO models are visually identical. Numbers and brain slices indicate the ICA components which act as network nodes. The width and colour of the connections indicate the strength of the partial correlations (red for positive correlations, blue for negative).

The group average functional connectivities inferred by the four models are very similar (too close to see major differences when connection strengths are displayed on a heat map). The (posterior mean) group-average partial correlation network for the sparse hierarchical model HIPPO is shown in Fig. 6.^18^ The posterior for the edge inclusion variables gave very high probabilities (over 99%) for all connections, presumably because the quantity of data in an hour's total recording time is sufficient to provide evidence for connectivity between all nodes, even if this connectivity is small in some cases. This point is explored further in the supplementary information, section C.3.

## Discussion

We have presented two hierarchical models for the functional connectivity measured with EEG, MEG or fMRI. One uses continuous priors to regularise the estimation of weak connectivities. The second explicitly promotes a sparse network structure, and provides posterior probabilities of a connection on each network edge. Both models characterise connectivity by the partial correlation between activations in ROIs, and jointly infer connection strengths for individual subjects and the population average. This ability to perform joint inference at both levels of the hierarchy, sharing information between subjects and regularising connection strengths towards the group mean, is an innovation in Bayesian covariance modelling, previously only possible for functions of linear effects (Gelman et al., 2014).

Accurate estimation of precision and covariance matrices is difficult and noisy. Most techniques designed to address this problem regularise weak elements of the matrices towards zero with some sparsity-promoting scheme. The importance of estimating precision matrices with some form of regularisation is clear in Fig. 2, where over many different datasets, even a simple approach like Tikhonov regularisation or GLASSO can reduce the reconstruction error by a third to a half. In simulations where we introduced some between-subject variability, the hierarchical Bayesian models outperform a wide range of methods that represent the state of the art in inverse covariance modelling. The non-Bayesian SGGM performs nearly as well. We note that this method has a very similar hierarchical structure to our ‘weakly sparse,’ continuous-prior Bayesian model, with terms designed to regularise subject connectivities towards the group and the group connectivities towards zero.

Hierarchical model structures and the partial pooling of information over subjects can be most useful when limited data are available within each subject (Gelman et al., 2014). Compare, for example, the improvement in matrix reconstruction for simulations 2–4, in Fig. 2, for which the number of subjects and network nodes remains constant, but the amount of data available within each subject increases. The hierarchical models do well in each case, but the differential improvement over more basic models is largest for the case with the least data. We observe the same effect in our studies of real data. Network estimation with a limited subset of both fMRI and MEG recordings can be greatly improved using the hierarchical models (see Fig. 4). The key point is that the quality of single-subject connectivity inference is enhanced, using commonalities between subjects to reduce the noise within each. Thus, estimates of the heritability of functional connectivity using meg beta-band data are increased (Fig. 5B), because a portion of the noisy variability within the dataset is reduced.

There are two areas in our results where the hierarchical models are not the top performers. Despite their success in the MEG heritability analyses, SGGM and the strongly sparse HIPPO model give mediocre results when applied to the limited subset of MEG recordings (Fig. 4B). However, meg networks are very noisy to estimate in comparison to fMRI (Colclough et al., 2016). The level of scan-to-scan variability may mean that the combination of three sessions that form our ‘ground truth’ is still not enough data to build a representative picture of each subject's functional connectivity, thereby skewing our results. The other area is in the quality of biological and behavioural trait prediction using functional networks estimated from fMRI data. It is possible that functional connectivity encodes very little information that can be extracted by a linear model about subjects' sex or their scores on picture vocabulary tests (Bijsterbosch et al., 2018). Alternatively, it may simply be that the quantity of data may be dominating the prior, such that the hierarchical model provides little improvement over simple estimates. In the HCP dataset, there are vastly more data available for inference in fMRI than for meg: the total recording time for fMRI is 1 h, for MEG 18 min, and in our analyses we estimate networks with 25 nodes in the former case and 39 in the latter. (Sampling rates are comparable across the two modalities because we apply our network models to the down-sampled power envelopes of MEG recordings.) We discuss this issue, and illustrate it with a simulated example, in supplementary section C.3.

Sparsity in functional connectivity matrices provides not only a mechanism to improve noisy estimates, but can also improve the interpretability of the networks. Our strongly sparse hierarchical model offers an analyst the ability to draw samples from the approximate posterior distribution of the graph representing the network structure of their dataset. They would then be able to construct posterior summaries of any function of that graph, f(Z). This idea was termed *Bayesian connectomics* when it was developed for structural connectivity by Janssen et al. in 2014. Using this fully probabilistic description of the network connections and their properties would be preferable to testing graph theory metrics (such as degree centrality or measures of ‘small world’ properties) over many binary network matrices created with a sliding scale of thresholds, as is currently common practice. However, while using MCMC chains to average over different models can provide effective regularisation of the parameter estimates, making inferences about graph theoretic functions of the network structure requires two conditions to be met. The first is basic, in that the analyst must be confident that they have run the sampling chains for long enough to have obtained a fair representation of the posterior. (George & McCulloch caution that the parameter space is so enormous that a sampler can at best ‘search for promising models, rather than compute the whole posterior.’) The second condition is that they must believe that a network model with a shared sparsity structure across subjects is a good representation of the data. We turn to this second assumption now.

The sparse hierarchical model we present expressly shares the sparsity structure over all subjects: the network structure is therefore considered a property of the entire population, about which no subject is considered to deviate. This may be plausible, particularly if analyses are restricted to sub-populations in which this assumption holds (fitting the model separately to patients and healthy controls, for example). However, it is also not clear that any sparsity in functional networks is an accurate biophysical assumption. We might expect some level of measurable connectivity between all brain regions, even if this level is small. There is support for this view from a recent tract-tracing study (Gămănut et al., 2018), and we note that inference using the large fMRI dataset gave evidence overwhelmingly in favour of the full model—that is, the model with all connections present. (This observation may however just be a consequence of the amount of data available, as discussed in supplementary section C.3 and Smith and Nichols, 2018.) We must also be cautious in drawing strong conclusions from the estimated graph structure, as other failures of our assumptions, such as of undirected network influences, linearity of the system or of network stationarity, may lead to over-confident identification of some connections in the sparse network. As a result, we suggest that the sparse model structure can be used for effective regularisation of connectivity estimation, but that further interpretation of the network structure be performed with care.

Which models can we recommend for connectivity estimation? A number are ruled out on inference time alone. The fused GLASSO and MGGM were not practical to run on our real-world examples; and the best Bayesian sparse model for individual connectivity estimation, the *G*-Wishart distribution, is impractical for use with moderate numbers of subjects, or even for single functional networks with 50 or more nodes. Between the strongly and weakly sparse versions of our HIPPO model, there was not a clear differentiator in terms of performance, or even in their ability to detect network edges (Fig. 3). However, in the weakly sparse model, the need for convergence of the posterior over the edge inclusion variables Z (which exhibit highly autocorrelated behaviour) is removed, so the total run time need not be as long. For example, the fMRI and MEG results were produced using parallel sampling chains that took about 14 h to run (for each chain) for the weakly sparse model, and 20 h for the sparse model. In comparison, the best non-Bayesian solution, SGGM, is very fast to run, although the search for optimal hyper-parameters using cross-validation can extend inference times to several hours on our datasets. sggm performs nearly as well in our simulations, and just as well in our real-data examples, as the Bayesian hierarchical models. For inference of individual subjects' connectivities, therefore, we would recommend either SGGM or our own HIPPO approach.

Our models do, however, pave the way for further development of connectivity modelling, and not just in the flexibility of the sparsity-promoting priors that can be accommodated. It would be simple to incorporate uncertainty over ROI time course estimation into our algorithm, using a Bayesian description of the parcellation process. Our approach could be further integrated with a more complex model in order to simultaneously infer both the parcellations and sparse functional networks, for example by extending the work of Harrison et al. (2015). Outside of neuroimaging, graphical model determination and covariance modelling are important techniques in financial analyses, protein network determination and gene expression modelling. Our hierarchical inference structure could also be applied to improve network estimation in these fields.

In conclusion, we have presented an advance in functional connectivity and inverse covariance modelling, by designing hierarchical Bayesian models for the distribution of connection strengths in subjects set within a wider group or population. We have demonstrated that hierarchical models, both our Bayesian approach and Ng et al.'s SGGM, are the best available choices for partial correlation models of functional networks. These models improve the quality of single-subject network estimates, particularly in small or noisy datasets, with concomitant increases in sensitivity to properties of interest (such as heritability) in the functional connectomes. Our Bayesian inference program, HIPPO, is sufficiently scalable to allow it to be applied to conventional neuroimaging datasets. The models are applicable both to fMRI and to MEG data, and we hope they will enable improved inference for studies in both modalities.

### Competing interests

S.S. is part-owner and shareholder of SBGneuro.

## Footnotes

1 OSL, the OHBA (Oxford Centre for Human Brain Activity) Software Library, and a Matlab implementation of our HIPPO algorithm are both available from www.github.com/ohba-analysis/.

2 The code used to perform this adjustment, nearestSPD.m was written by John D'Errico and is available from uk.mathworks.com/matlabcentral/fileexchange/42885-nearestspd.

3 Both of these matrices are available from the Brain Connectivity Toolbox, at sites.google.com/site/bctnet/datasets.

4 Python routines for solving this problem are available as part of nilearn. See nilearn.github.io/connectivity for more details.

5 R code for solving Danaher et al.'s group GLASSO and fused GLASSO using the alternating direction method of multipliers algorithm is available as the package JGL, from cran.r-project.org/web/packages/JGL/index.html.

6 Matlab code for solving the model was obtained from the authors.

7 If Ω is (*G*-) Wishart distributed, $\Omega ∼W_{G}\left(\delta ,V\right)$, then its expectation is 〈Ω〉=δV.

8 BayesGlasso is available from msu.edu/∼haowang/RESEARCH/Bglasso/bglasso.html.

9 BayesGGM_SSVS is available from msu.edu/∼haowang/.

10 ggm_gwish_cbf_direct.m is available from github.com/ccnlab/BaCon/tree/master/ggm.

11 Software implementing the method is available from odin.mdacc.tmc.edu/∼cbpeterson/software.html.

12 db.humanconnectome.org.

13 These two confounds are excluded when sex is the predicted variable.

14 Junyang Qian's Matlab implementation of Friedman et al.'s Glmnet algorithms is available from web.stanford.edu/∼hastie/glmnet_matlab/.

15 The Advanced Permutation inference for ACE models software is available from warwick.ac.uk/tenichols/apace.

16 Available at db.humanconnectome.org/megatrawl/.

17 *Crystallised intelligence* is defined by Wikipedia (on the 21^st^ August 2016) as ‘the ability to use skills, knowledge, and experience. It does not equate to memory, but it does rely on accessing information from long-term memory.’ This is in contrast to *fluid intelligence,* which characterises an ability to reason, deduce and to solve novel problems.

18 Figure produced using Paul McCarthy's visualisation tool in FSLNets.

Appendix A Supplementary data related to this article can be found at https://doi.org/10.1016/j.neuroimage.2018.04.077.
