# Supplementary material for: Multi-subject hierarchical inverse covariance modelling improves estimation of functional brain networks
Source: Neuroimage. 2018 Sep;178:370–84. doi: 10.1016/j.neuroimage.2018.04.077 (PMC6565932; doi:10.1016/j.neuroimage.2018.04.077)
Supplement: Supplementary data [file mmc1.pdf]

Supplementary information for 'Multi-subject hierarchical inverse covariance modelling improves estimation of functional brain networks'

GILES L. COLCLOUGH,[1,2,3] MARK W. WOOLRICH,[*1,2] SAMUEL J. HARRISON,[1,2]
PEDRO A. ROJAS LÓPEZ,[4] PEDRO A. VALDES-SOSA,[5,4] AND STEPHEN M. SMITH[2]

[1]*Oxford Centre for Human Brain Activity (OHBA), Wellcome Centre for Integrative Neuroimaging, Department of Psychiatry, University of Oxford, Oxford, UK.*
[2]*Oxford centre for Functional MRI of the Brain (FMRIB), Wellcome Centre for Integrative Neuroimaging, Nuffield Department of Clinical Neurosciences, University of Oxford, Oxford, UK.*
[3]*Centre for Doctoral Training in Healthcare Innovation, Institute of Biomedical Engineering Science, Department of Engineering, University of Oxford, Oxford, UK.*
[4]*Neuroinformatics Department, El Centro de Neurociencias de Cuba (CNEURO), La Habana, Cuba.*
[5]*The Clinical Hospital of Chengdu Brain Science Institute, MOE Key Lab for Neuroinformation, University of Electronic Science and Technology of China, Chengdu, China.*

[*]Corresponding email address: mark.woolrich@ohba.ox.ac.uk.

A Inference for the hierarchical model

Full details of the inference procedure and Markov chain Monte Carlo (MCMC) sampling scheme for the hierarchical Bayesian models of functional connectivity are given here. The conditional distributions for the sampler are derived in supplementary information B and the full sampling algorithm is given in algorithm 2.

Inference occurs in two stages. First, estimation of a time course within each region of interest (ROI). Second, inferring the precision matrices for each subject. Welding these two stages together into a joint inference procedure is a level of complexity that we have not yet tackled.

A.1 GENERATIVE MODEL FOR RESTING-STATE ACTIVATION

A.1.1 Description of activation within regions of interest

We are interested in describing the connectivity between several regions of interest over the brain. Given temporally demeaned activations $\boldsymbol{X} \in \mathbb{R}^{v \times n_s}$ in each of N subjects s, defined over a grid of v voxels or points on a cortical surface, and sampled at n_s time points, we can estimate a single time course for each of p ROIs using a spatial basis set, a linear weighting over voxels,

$$\boldsymbol{X}_s = \boldsymbol{A}\boldsymbol{Y}_s + \boldsymbol{E}; \quad \boldsymbol{E} \sim \mathcal{N}\left(\boldsymbol{0}, \boldsymbol{\Sigma}_e\right). \tag{1}$$

Here, $\boldsymbol{Y}_s = \left[\boldsymbol{y}_1^s, \boldsymbol{y}_2^s, \ldots \boldsymbol{y}_{n_s}^s\right] \in \mathbb{R}^{p \times n_s}$ represents the activation within ROIs, $\boldsymbol{A} \in \mathbb{R}^{v \times p}$ is the spatial basis set (which may simply be binary region allocations, or, for example, a spatial map derived from independent component analysis (ICA)), and $\boldsymbol{E}$ represents additive noise with covariance structure $\boldsymbol{\Sigma}_e$ over voxels.

We use the general term *activation* to encompass changes in blood-oxygenation-level dependent (BOLD) response over time in functional magnetic resonance imaging (fMRI), or fluctuations in the power envelope of oscillatory activity measured with magnetoencephalography (MEG).

Uncertainty about the ROI time courses in the first phase can be easily passed up to the second by drawing consecutive samples of the sum-of-squares matrix $\boldsymbol{S}$ for each subject, and using these as the input for each step of the MCMC algorithm (Gelman *et al.*, 2014).

To illustrate this point, we describe a simple linear regression model to infer the activation within each ROI (1). Assuming for simplicity that the measurement noise in the voxels is isotropic and uncorrelated, sharing a common variance σ^2, and using simple uninformative priors,

$$\left(\boldsymbol{Y}, \sigma^2\right) \sim 1/\sigma^2 \tag{2}$$

(Gelman *et al.*, 2014, ch. 14), the posterior is easily found as

$$\begin{aligned}
\boldsymbol{Y}|\sigma^2, \boldsymbol{X} &\sim \mathcal{N}\left(\hat{\boldsymbol{Y}};\, \boldsymbol{V}\sigma^2\right) \\
\sigma^2|\boldsymbol{X} &\sim \text{Inv-}\chi^2\left(\left(v-p\right)n_s,\, s^2\right) \\
\hat{\boldsymbol{Y}} &= \left(\boldsymbol{A}'\boldsymbol{A}\right)^{-1}\boldsymbol{A}'\boldsymbol{X} \\
\boldsymbol{V} &= \left(\boldsymbol{A}'\boldsymbol{A}\right)^{-1} \\
s^2 &= \frac{1}{\left(v-p\right)n_s}\,\text{trace}\left(\left(\boldsymbol{X}-\boldsymbol{A}\hat{\boldsymbol{Y}}\right)\left(\boldsymbol{X}-\boldsymbol{A}\hat{\boldsymbol{Y}}\right)'\right),
\end{aligned} \tag{3}$$

and samples for the sum-of-squares matrix for demeaned $\boldsymbol{Y}$ can be easily drawn, conditional on draws of σ, from a Wishart distribution,

$$\boldsymbol{S} \sim \mathcal{W}_p\left(n_s, \boldsymbol{V}\sigma^2\right). \tag{4}$$

It would also be possible to use inference procedures that can account for more complicated or correlated noise models (e.g.Chappell *et al.*, 2009; Penny *et al.*, 2007). However, as the best approach for constructing representative time courses for ROIs is still a matter of much debate (Beckmann *et al.*, 2009; Calhoun *et al.*, 2001; Filippini *et al.*, 2009; Harrison *et al.*, 2015), and we prefer to focus here on the precision modelling, we opt to use a simple linear regression point-estimate, $\hat{Y}_s = \left(A^\dagger A\right)^{-1} A^\dagger X_s$.

A.1.2 Estimation of temporal degrees of freedom

Our model for functional connectivity ignores the temporal complexity of the data, assuming that all samples are independently drawn from the Gaussian graphical model (GGM) structure. However, we cannot ignore the fact that the data we describe have strong temporal autocorrelations, such that not every sample provides new information, independent from its predecessors. Using a poor (over-) estimate of the number of independent samples in the data will cause the Bayesian model to inadequately capture the appropriate level of uncertainty within the posterior distribution.

One approach to dealing with autocorrelated input data would be to temporally whiten the data, but the merits of fitting an auto-regressive model then modelling the covariance structure of the residuals have not been investigated. Motivated partly by the estimation of spatial degrees of freedom in functional neuroimaging studies (Groves *et al.*, 2011; Worsley *et al.*, 1992) and partly by the estimation of the effective sample size of an MCMC chain, we estimate the number of independent data samples in each subject as

$$n_s = \frac{n}{1 + 2\sum_{t=1}^{\infty} \rho_t}\,, \tag{5}$$

where n indicates the number of data points and ρ_t is the temporal autocorrelation of an ROI time course at lag t. We estimate this effective sample size in each ROI, then simply average over ROIs to get the final estimate. See Gelman *et al.* (2014), ch. 11, for a full discussion in the context of Monte-Carlo sampling and computation of the autocorrelations.

A.1.3 Restatement of the model

The likelihood of the ROI activation data is given in equation (1) in the main text as

$$Y_s \sim \mathcal{N}\left(\mathbf{0}, \boldsymbol{\Omega}_s^{-1}\right), \ \boldsymbol{\Omega}_s \in \mathbb{P}_{\mathcal{G}}\,.$$

The prior for the strongly sparse model, set out in equations (3), (9) and (10) in the main text is

$$\begin{aligned}
\pi\left(\boldsymbol{\Omega}_s\right) &= \prod_{i=1}^{p} \operatorname{Exp}\left(\omega_{ii}^s;\ \frac{\lambda^s}{2}\right) \prod_{i<j} \pi\left(\omega_{ij}^s;\ \sigma_{ij}, \mu_{ij}, z_{ij}\right) \pi\left(\lambda^s\right) \qquad (6)\\
\lambda^s &\sim \operatorname{Ga}\left(1/3, 0\right)\\
\left(\omega_{ij}^s | \sigma_{ij}, \mu_{ij}, z_{ij} = 1\right) &\sim \mathcal{N}\left(\mu_{ij}, \sigma_{ij}^2\right)\\
\left(\omega_{ij}^s | z_{ij} = 0\right) &\sim \delta_0\\
\log \sigma_{ij} &\sim \mathcal{N}\left(\log m_\sigma, s_\sigma^2\right)\\
\mu_{ij} &\sim \mathcal{N}\left(0, \chi^2\right)\\
\chi &\sim \mathcal{C}^+\left(0, A\right)\\
z_{ij} &\sim \operatorname{Bernoulli}\left(a\right)\\
a &\sim \operatorname{Beta}\left(a_\pi, b_\pi\right)\,.
\end{aligned}$$

The weakly-sparse model is simply formed by removing the spike-and-slab distribution, setting $a = 1$ and $z_{ij} = 1\,\forall\, i, j$.

A.1.4 *Expanding the Cauchy distribution*

The prior on μ_{ij} is complex, but can be re-written as a scale mixture of normals using a simple parameter expansion. Usefully, this change of variables creates a conditionally-conjugate prior structure, simplifying the inference procedure and permitting marginalisation over μ_{ij} to collapse part of the Gibbs sampler (van Dyk & Park, 2008). Following the steps in Gelman (2006); Gelman *et al.* (2008, 2014); and Polson & Scott (2012), we obtain

$$\begin{aligned} \mu_{ij} &= \xi \eta_{ij} \\ \eta_{ij} &\sim \mathcal{N}\left(0, \sigma_\eta^2\right) \\ \sigma_\eta^2 &\sim \text{Inv-}\chi^2\left(1, A\right) \\ \xi &\sim \mathcal{N}\left(0, 1\right) \\ \chi &= |\xi|\sigma_\eta \,. \end{aligned} \tag{7}$$

Redundant parameter expansion in this manner was originally designed to improve the convergence of the Expectation Maximisation algorithm (Liu *et al.*, 1998), then used to increase the movement available to Gibbs samplers (Gelman *et al.*, 2008). It has since been used to create a wide range of flexible priors, in addition to that used here (Carvalho *et al.*, 2010; Gelman *et al.*, 2014; Peltola *et al.*, 2012). It is not necessary, when sampling, for ξ and $\boldsymbol{\eta}$ to converge, only for the meaningful parameters χ and $\boldsymbol{\mu}$.

A.1.5 *Joint probability distribution*

We are now in a position to write out the full joint probability distribution of the parameters and data,

$$\begin{aligned} p\left(\{\boldsymbol{\Omega}_s\}, \boldsymbol{Z}, \boldsymbol{\mu}, \{\lambda^s\}, \boldsymbol{\sigma}, \chi, a, \{\boldsymbol{Y}_s\}\right) \propto \prod_s^N \Bigg\{ & \frac{|\boldsymbol{\Omega}_s|^{\frac{n_s}{2}}}{(2\pi)^{\frac{pn_s}{2}}} e^{-\frac{1}{2}\,\text{trace}[S\boldsymbol{\Omega}_s]} \prod_{i=1}^{p} \frac{\lambda^s}{2} e^{-\frac{\lambda^s}{2}\omega_{ii}^s} \\ & \times \prod_{i<j} \left\{ \left(\frac{1}{\left(2\pi\sigma_{ij}^2\right)^{1/2}} e^{-\frac{\left(\omega_{ij}^s - \xi\eta_{ij}\right)^2}{2\sigma_{ij}^2}} \right)^{z_{ij}} \right. \\ & \qquad \left. \times\, \delta\left(\omega_{ij}^s\right)^{\left(1-z_{ij}\right)} \right\} \\ & \times \frac{\lambda^{s\,1/3-1}}{\Gamma\left(1/3\right)} \Bigg\} \\ & \times \prod_{i<j} \left\{ a^{z_{ij}} \left(1-a\right)^{\left(1-z_{ij}\right)} \frac{1}{\left(2\pi\sigma_\eta^2\right)^{\frac{1}{2}}} e^{-\frac{\eta_{ij}^2}{2\sigma_\eta^2}} \right\} \\ & \times \prod_{i<j} \left\{ \frac{1}{\sigma_{ij}\left(2\pi s_\sigma^2\right)^{\frac{1}{2}}} e^{-\frac{\left(\log\sigma_{ij} - \log m_\sigma\right)^2}{2s_\sigma^2}} \right\} \\ & \times \frac{a^{a_\pi-1}\left(1-a\right)^{b_\pi-1}}{\mathrm{B}\left(a_\pi, b_\pi\right)} \frac{1}{\sqrt{2\pi}} e^{-\xi^2} \frac{\left(A/2\right)^{1/2}}{\Gamma\left(1/2\right)} \frac{e^{-\frac{A}{2\sigma_\eta^2}}}{\sigma_\eta^{2^{1+\frac{1}{2}}}} \,. \end{aligned} \tag{8}$$

A.2 MCMC SAMPLING SCHEME

A.2.1 MCMC as the tool of choice

In the strongly sparse hierarchical model, we used a true Dirac delta function model to impose shared sparsity over subjects. This approach is in contrast to that of Wang (2015), which approximates the spike with a narrow-width Gaussian. The benefits of using a delta function include the explicit selection of variables and the ability to average over different model structures; a reduction of computational complexity for increasingly sparse models; insensitivity of inference to hyperparameters controlling the width of the spike and slab; and adaptive shrinkage which can leave untouched variables with strong support in the data, but strongly regularise those of more dubious merit. The downside is the scaling of the problem. For a regression model with k variables, or a network matrix with p nodes and $k = \frac{p(p-1)}{2}$ links, there are 2^k possible models to consider. For example, a network with 30 nodes has about 10^{131} possible models! A variational inference approach, with a posterior that factorises into columns, did not seem feasible. MCMC samplers, then, are the tool of choice for approximate inference, but come with a health warning. The scale of the inference problem will likely be so large that 'the sampler is used to search for promising models, rather than compute the whole posterior' (George & McCulloch, 1997). Converging rapidly to a set of high-probability states is, however, a task at which MCMC chains can excel.

A.2.2 Description of approach

We propose a Metropolis-within-collapsed-Gibbs (MWCG) sampler for inference of the precision matrices. The basic structure is of conditional Gibbs draws of all parameters that can be identified with particular columns of the matrices. A sample consists of drawing from each column in turn, in a random order. Because of the symmetry within the matrices, this means that these parameters are actually drawn twice within one sample. Some parameters are common to all columns, and are drawn from their conditional distributions separately.

Within each Gibbs draw for a column, the model is largely conditionally conjugate, and many parameters can be marginalised out, collapsing the Gibbs sampler for this subset of parameters (van Dyk & Park, 2008). At the top level, an Metropolis-Hastings (MH) algorithm is used to sample Z, the variables identifying which edges are included in the model. The MH sampler is able to explore a conditional distribution which marginalises over all subject-level edge strengths, and the group mean edge strength, substantially reducing the correlations between successive draws. It can be considered as a Bayesian model averaging procedure, in which the sampler accepts or rejects proposed models based on the model evidence (within a column) associated with their particular structure. The group-average connectivity strengths $\boldsymbol{\mu}$ can be drawn conditional on Z, marginalised over the subject-level strengths, which themselves fall out as Gibbs steps at the bottom of the hierarchy.

A.2.3 Edge-allocation vectors

We infer edge inclusion, for each column of the precision matrices, using a Metropolis-Hastings chain. Following ideas in Peltola *et al.* (2012), we propose changing k edges on each proposal, drawing k from a truncated geometric distribution—a geometric distribution has only one tunable parameter, simplifying the optimisation of the sampler. The proposal consists of deciding in turn, for each of the k edges, whether to add or remove a variable, then picking the altered variable at random. The acceptance probability corresponds to the ratio of model evidences for the posterior with the set of new variables to the set of old variables. The model evidence is computed by averaging out $\boldsymbol{u}^s$ and $\boldsymbol{\mu}_{12}$.

Move proposal A move of k edges is proposed from a geometric distribution with parameter $l \in (0, 1]$, $p(k) = (1 - l)^{k-1} l$, which has a mode at 1, and which is truncated at either 20 or the number of edges

in the column, whichever is smaller. Variables are proposed to be added or removed from the model with a probability of 1/2, until there are no more variables to add or remove, when the probability becomes 1 in the remaining direction, until k proposals have been made. Taking $\boldsymbol{\gamma}$ to be the edge inclusion variables for a particular column, $\boldsymbol{\gamma} = z_{12}$, we call the proposed model $\boldsymbol{\gamma}^\star$. Borrowing the notation from Peltola *et al.* (2012), we call the sequence of variable changes in the proposal $\boldsymbol{\zeta}^\star = [\zeta_1^\star \dots \zeta_k^\star]$, such that $\boldsymbol{\gamma}^\star$ is produced by applying to $\boldsymbol{\gamma}$ flips of the variables contained in $\boldsymbol{\zeta}^\star$. The proposal distribution for each variable to add, conditional on the previous changes, is given by the normalised edge inclusion probabilities of all variables not in $\boldsymbol{\gamma}$ or already proposed in $\boldsymbol{\zeta}^\star$. We estimate edge inclusion probabilities m_j from MCMC samples in the warm-up phase, bounding from below at 0.1 and from above at 0.9. The proposal distribution for variables to remove uses the same process, with normalised estimates of the edge exclusion probabilities $1 - m_j$ over the variables in $\boldsymbol{\gamma}$ and not already proposed in $\boldsymbol{\zeta}^\star$. We further define $\boldsymbol{\zeta}$ to be the set of edge changes which exactly undoes the processes in $\boldsymbol{\zeta}^\star$, to take $\boldsymbol{\gamma}^\star$ back to $\boldsymbol{\gamma}$. The full proposal distribution, $q_1\left(\boldsymbol{\gamma}^\star|\boldsymbol{\gamma}\right)$, is a sequential product of the individual proposals,

$$
\begin{aligned}
q_1\left(\boldsymbol{\gamma}^\star|\boldsymbol{\gamma},\boldsymbol{\zeta}^\star\right) &= \begin{cases} 1 & \text{if } \boldsymbol{\gamma}^\star \text{ incorporates changes } \boldsymbol{\zeta}^\star \text{ to } \boldsymbol{\gamma} \\ 0 & \text{otherwise} \end{cases} \\
q_1\left(\boldsymbol{\zeta}^\star|\boldsymbol{\gamma}\right) &= \prod_{r=1}^{k} q_1\left(\zeta_r^\star|\boldsymbol{\gamma},\zeta_{1:r-1}^\star\right) \\
q_1\left(\zeta_r^\star = +j|\boldsymbol{\gamma},\zeta_{1:r-1}^\star\right) &= \frac{1}{2}\frac{m_j}{\sum_{s\notin\left(\boldsymbol{\gamma}\cup\zeta_{1:r-1}^\star\right)} m_s} \\
q_1\left(\zeta_r^\star = -j|\boldsymbol{\gamma},\zeta_{1:r-1}^\star\right) &= \frac{1}{2}\frac{1-m_j}{\sum_{s\in\left(\boldsymbol{\gamma}\setminus\zeta_{1:r-1}^\star\right)} 1-m_s} \\
h_1\left(\boldsymbol{\zeta}|\boldsymbol{\zeta}^\star,\boldsymbol{\gamma},\boldsymbol{\gamma}^\star\right) &= \begin{cases} 1 & \text{if } \boldsymbol{\zeta} \text{ is constructed using algorithm 1} \\ 0 & \text{otherwise.} \end{cases}
\end{aligned}
\tag{9}
$$

Algorithm 1: PROCEDURE FOR GENERATING REVERSE PROPOSALS. This algorithm, designed by Peltola *et al.*, is used to construct ζ and $\zeta^{\star\prime}$, the set of steps to undo proposals $\boldsymbol{\gamma}^{\star}$ and $\boldsymbol{\gamma}'$. The algorithm is designed such that it is reversible (generating ζ, then reversing to generate $\zeta^{\star}$, returns to the initial proposal).

```
def form_backward_move_proposal(zetaS, gamma,  gammaS):
    ## 1.
    # Construct intermediate
    zeta_b = zetaS
    zeta_b = reverse_order_of_removals(zeta_b)
    zeta_b = set_all_operations_to_additions(zeta_b)

    ## 2.
    # convert elements to removals to make
    # a valid proposal from gammaS
    zeta = make_valid(zeta_b, gammaS)
    zeta = reverse_order_of_removals(zeta)

    return zeta
```

Acceptance probability The jump ratio and acceptance probability for a proposal $(\boldsymbol{\gamma}^{\star}, \zeta^{\star}, \zeta)$ are

$$\begin{aligned} r &= \frac{p\left(\boldsymbol{\gamma}^{\star} | \{Y_s\}, \chi, \boldsymbol{\sigma}, a\right) q_1\left(\boldsymbol{\gamma}, \zeta | \boldsymbol{\gamma}^{\star}\right) p(k)\, h_1\left(\zeta^{\star} | \zeta, \boldsymbol{\gamma}^{\star}, \boldsymbol{\gamma}\right)}{p\left(\boldsymbol{\gamma} | \{Y_s\}, \chi, \boldsymbol{\sigma}, a\right) q_1\left(\boldsymbol{\gamma}^{\star}, \zeta^{\star} | \boldsymbol{\gamma}\right) p(k)\, h_1\left(\zeta | \zeta^{\star}, \boldsymbol{\gamma}, \boldsymbol{\gamma}^{\star}\right)} \\ &= \frac{p\left(\boldsymbol{\gamma}^{\star} | \{Y_s\}, \chi, \boldsymbol{\sigma}, a\right) q_1\left(\boldsymbol{\gamma} | \zeta, \boldsymbol{\gamma}^{\star}\right) q_1\left(\zeta | \boldsymbol{\gamma}^{\star}\right) h_1\left(\zeta^{\star} | \zeta, \boldsymbol{\gamma}^{\star}, \boldsymbol{\gamma}\right)}{p\left(\boldsymbol{\gamma} | \{Y_s\}, \chi, \boldsymbol{\sigma}, a\right) q_1\left(\boldsymbol{\gamma}^{\star} | \boldsymbol{\gamma}, \zeta^{\star}\right) q_1\left(\zeta^{\star} | \boldsymbol{\gamma}\right) h_1\left(\zeta | \zeta^{\star}, \boldsymbol{\gamma}, \boldsymbol{\gamma}^{\star}\right)}, \\ a_1\left(\boldsymbol{\gamma}^{\star} | \zeta^{\star}, \zeta, \boldsymbol{\gamma}\right) &= \min(1, r)\ , \end{aligned} \tag{10}$$

and the evidence for model $\boldsymbol{\gamma}$ is derived in supplementary section B as

$$\begin{aligned} p\left(\boldsymbol{\gamma} | \{Y_s\}, \chi, \boldsymbol{\sigma}_{12}, a\right) \propto & \frac{1}{(2\pi)^{\sum_s n_s/2}} |D_\tau^+|^{N/2} a^{|\gamma_+|} (1-a)^{|\gamma_-|} \\ & \times \prod_s \left\{ |\boldsymbol{\Delta}_+^s|^{1/2} e^{\frac{1}{2} S_{12}^{+\dagger} \boldsymbol{\Delta}_+^s S_{12}^+} \right\} \frac{|\boldsymbol{\Xi}|^{1/2}}{\chi^{2^{|\gamma_+|/2}}} e^{\frac{1}{2} \boldsymbol{\epsilon}_+^\dagger \boldsymbol{\Xi} \boldsymbol{\epsilon}_+}\ , \end{aligned} \tag{11}$$

where symbols $+$ and $-$ denote the subsets of variables within the column which are included or excluded, respectively, in the model; $|\gamma_+|$ is the number of included variables in the column; and the remaining variables are defined as

$$\boldsymbol{\tau} = \boldsymbol{\sigma}_{12}^{-2} \tag{12}$$

$$D_\tau = \operatorname{diag}\left(\boldsymbol{\sigma}_{12}^{-2}\right) \tag{13}$$

$$\boldsymbol{\rho}_+^s = \left(D_\tau^+ \boldsymbol{\mu}_+ - S_{12}^+\right) \tag{14}$$

$$\boldsymbol{\Delta}_+^s = \left(\boldsymbol{\Upsilon}_+^s + D_\tau^+\right)^{-1} \tag{15}$$

$$\boldsymbol{\Xi} = \left(\frac{1}{\chi^2} + N D_\tau^+ - \sum_s D_\tau^+ \boldsymbol{\Delta}_+^s D_\tau^+\right)^{-1} \tag{16}$$

$$\boldsymbol{\epsilon}_+^\dagger = \sum_s S_{12}^{+\dagger} \boldsymbol{\Delta}_+^s D_\tau^+\ . \tag{17}$$

An MH update with this acceptance probability will converge to the target distribution (Peltola *et al.*, 2012; Storvik, 2011).

Delayed rejection scheme The sampler outlined above can be very inefficient in exploring the parameter space, with only 1-10 edge changes in 100 samples on real data. To improve on this, we use an additional technique suggested for sparse linear regression by Peltola *et al.* (2012): the ability to create a second proposal, after the first is rejected, which is in some way conditional on the first but which uses fewer computations to propose and test for acceptance than the creation of an entirely new proposal. Delayed rejection (Green & Mira, 2001; Mira, 2001; Trias *et al.*, 2009) is this process of proposing a secondary move, in such a fashion that detailed balance of the whole chain is preserved. Peltola *et al.* demonstrate that a secondary move can be constructed which is guaranteed to be accepted. The proposal distribution for this second move picks from a small number of models (possible choices for the vector $\boldsymbol{\gamma}'$), which include the original model $\boldsymbol{\gamma}$. We describe this process now.

If the first proposal $\boldsymbol{\gamma}^\star$ is rejected, which occurs with probability $1-a_1$, a second proposal $\boldsymbol{\gamma}'$ is created. If k edges were first proposed to be altered, by applying $\boldsymbol{\zeta}^\star$ to $\boldsymbol{\gamma}$, $\boldsymbol{\gamma}'$ is chosen from the 2^k models that consist of applying subsets of $\boldsymbol{\zeta}^\star$ to $\boldsymbol{\gamma}$. Thus if $\boldsymbol{\gamma} = [1100]$ and the first proposal was $\boldsymbol{\zeta}^\star = \{+3, +4, -1\}$ to form $\boldsymbol{\gamma}^\star = [0111]$, the second proposal must be formed by applying one of $\boldsymbol{\zeta}'$: { {}, {+3}, {+4}, {−1}, {+3, +4}, {+3, −1}, {+4, −1}, {+3, +4, −1} } to $\boldsymbol{\gamma}$: $\boldsymbol{\gamma}'$ = { [1100], [1110], [1101], [0100], [1111], [0110], [0101], [0111] }. This set of models *includes* the original model $\boldsymbol{\gamma}$ and the rejected first proposal $\boldsymbol{\gamma}^\star$.

As the delayed rejection proposal is a *two-step* move, in order to satisfy reversibility we must be able to construct a two-step backwards move, with proposal distribution $q_2\left(\boldsymbol{\gamma}|\boldsymbol{\gamma}', \boldsymbol{\zeta}^{\star\prime}\right)$. This requires its own intermediate proposal, $\boldsymbol{\gamma}^{\star\prime}$, which need not be the same as $\boldsymbol{\gamma}^\star$, but should require the same number of edge flips to reach, k, as the initial proposal.

The proposal distribution q_2 for choosing between this set of models is constructed such that the acceptance probability of this delayed step is unity, $a_2 = 1$. We can see that the full expression for the acceptance probability after a two-stage proposal is

$$a_2\left(\boldsymbol{\gamma}^\star|\boldsymbol{\zeta}^\star, \boldsymbol{\zeta}, \boldsymbol{\gamma}\right) = \min\left(1, \frac{p\left(\boldsymbol{\gamma}'|\{Y_s\}, \chi, \boldsymbol{\sigma}, a\right) q_1\left(\boldsymbol{\gamma}^{\star\prime}, \boldsymbol{\zeta}^{\star\prime}|\boldsymbol{\gamma}'\right) p\left(k\right)}{p\left(\boldsymbol{\gamma}|\{Y_s\}, \chi, \boldsymbol{\sigma}, a\right) q_1\left(\boldsymbol{\gamma}^\star, \boldsymbol{\zeta}^\star|\boldsymbol{\gamma}\right) p\left(k\right)} \times \frac{\left(1 - a_1\left(\boldsymbol{\gamma}^{\star\prime}|\boldsymbol{\zeta}^{\star\prime}, \boldsymbol{\zeta}', \boldsymbol{\gamma}'\right)\right) q_2\left(\boldsymbol{\gamma}|\boldsymbol{\gamma}', \boldsymbol{\zeta}^{\star\prime}\right)}{\left(1 - a_1\left(\boldsymbol{\gamma}^\star|\boldsymbol{\zeta}^\star, \boldsymbol{\zeta}, \boldsymbol{\gamma}\right)\right) q_2\left(\boldsymbol{\gamma}'|\boldsymbol{\gamma}, \boldsymbol{\zeta}^\star\right)}\right), \tag{18}$$

where as before, $\boldsymbol{\zeta}^{\star\prime}$ is constructed using algorithm 1. The secondary proposal is therefore guaranteed to be accepted for

$$q_2\left(\boldsymbol{\gamma}'|\boldsymbol{\gamma}, \boldsymbol{\zeta}^\star\right) \propto p\left(\boldsymbol{\gamma}'|\{Y_s\}, \chi, \boldsymbol{\sigma}, a\right) q_1\left(\boldsymbol{\gamma}^{\star\prime}, \boldsymbol{\zeta}^{\star\prime}|\boldsymbol{\gamma}'\right) \times p\left(k\right)\left(1 - a_1\left(\boldsymbol{\gamma}^{\star\prime}|\boldsymbol{\zeta}^{\star\prime}, \boldsymbol{\zeta}', \boldsymbol{\gamma}'\right)\right), \tag{19}$$

which is the product of the probability of the target model, $\boldsymbol{\gamma}'$, the jumping probability to the intermediate proposal in the reverse direction, and the probability of rejecting that proposal. The distribution is normalised over the range of models considered for $\{\boldsymbol{\gamma}'\}$. Sampling from q_2 involves re-computing (11) with sequential updates to a set of Cholesky decompositions as variables are added or removed. It is possible to do this with an efficient implementation, in which the computational complexity scales as $\mathcal{O}\left(N2^k k\right)$; see Clarke (1981); Peltola *et al.* (2012); and Smith (1989) for details.

Utilising this delayed rejection scheme can greatly increase the efficiency on real data, realising around 25 edge changes in 100 samples, an improvement of two- to ten-fold on performance without it.

Construction of reverse move We use the construction of a backward proposal set out in Peltola *et al.* (2012) to build $\zeta^{\star\prime}$ and, for consistency, ζ. The algorithm (1) is designed such that it is reversible (generating ζ , then reversing to generate $\zeta^{\star}$, returns to the initial proposal), and such that $q_2\left(\gamma'|\gamma,\zeta^{\star}\right) = q_2\left(\gamma'|\gamma',\zeta^{\star\prime}\right)$, which ensures that the probabilities in the distribution of the delayed proposal are independent of which primary model they are conditioned upon.

A.2.4 Between-subject variance

Within each column, we sample the between-subject variance parameters $\boldsymbol{\sigma}_{12}$ using an MH kernel. A Gibbs draw conditional on the subject-level connection strengths is possible, but we found that this led to highly correlated behaviour.

The target density is derived in supplementary section B,

$$\begin{aligned} p\left(\boldsymbol{\sigma}_{12}|\chi,\boldsymbol{\gamma},\boldsymbol{\mu}_{12},\{Y_s\}\right) \propto & \frac{1}{(2\pi)^{\sum_s n_s/2}} |D_\tau^+|^{N/2} e^{-\frac{N}{2}\boldsymbol{\mu}_+^\dagger D_\tau^+ \boldsymbol{\mu}_+} \\ & \times \prod_s \left\{ |\boldsymbol{\Delta}_+^s|^{1/2} e^{\frac{1}{2}\boldsymbol{\rho}_+^{s\dagger}\boldsymbol{\Delta}_+^s \boldsymbol{\rho}_+^s} \right\} \\ & \times \prod_i \left\{ \frac{1}{\sigma_{i\cdot}\left(2\pi s_\sigma^2\right)^{\frac{1}{2}}} e^{-\frac{(\log\sigma_{i\cdot} - \log m_\sigma)^2}{2 s_\sigma^2}} \right\} . \end{aligned} \tag{20}$$

We use a proposal density which is multivariate normal on $\log\boldsymbol{\sigma}_{12}$,

$$\begin{aligned} q\left(\boldsymbol{\sigma}'|\boldsymbol{\sigma}\right) = & \frac{1}{\prod_i \sigma_{i\cdot}\left(2\pi\right)^{q/2}} |c^2 \boldsymbol{\Sigma}_{\log\sigma}|^{-1/2} \\ & \times e^{-\frac{1}{2}(\log\boldsymbol{\sigma} - \log\boldsymbol{\sigma}')^T \left(c^2\boldsymbol{\Sigma}_{\log\sigma}\right)^{-1} (\log\boldsymbol{\sigma} - \log\boldsymbol{\sigma}')} , \end{aligned} \tag{21}$$

with a scaling on the covariance matrix of c^2. This gives an acceptance probability of

$$a\left(\boldsymbol{\sigma}_{12}'|\boldsymbol{\sigma}_{12}\right) = \min\left(1, \frac{p\left(\boldsymbol{\sigma}_{12}'|\chi,\boldsymbol{\gamma},\boldsymbol{\mu}_{12},\{Y_s\}\right)}{p\left(\boldsymbol{\sigma}_{12}|\chi,\boldsymbol{\gamma},\boldsymbol{\mu}_{12},\{Y_s\}\right)} \frac{\prod_i \sigma_{i\cdot}}{\prod_i \sigma_{i\cdot}'}\right) . \tag{22}$$

A.2.5 Adaptive sampling phase

We use a *warm-up period* (an initial set of samples, often the first half) to automatically adapt the properties of our sampler. At the end of the warm-up, we fix all values and let the sampler continue into the *sampling phase* proper. Adjusting the properties of the kernel during the warm-up breaks the detailed balance conditions of the MCMC chain, but this is not problematic if the samples from the warm-up period are not used for inference (Gelman *et al.*, 2014). Four parameters are adapted. For the proposals on $\boldsymbol{Z}$, the edge inclusion probabilities $\boldsymbol{M}$ and the parameter for the geometric distribution which controls how many edges are proposed, l, are updated. For the proposals on the between-subject variance parameters, $\boldsymbol{\sigma}$, the covariance matrix and scale of the kernel are adjusted.

The covariance structure of $\log\boldsymbol{\sigma}$ and the edge inclusion probabilities can be periodically updated using the history of samples in the warm-up period. To tune l and c, however, a criterion for the optimal acceptance rate of the respective MH samplers is needed. Various authors propose different heuristics for this target. David MacKay (MacKay, 2003) motivates a rule-of-thumb acceptance rate of 0.5 as maximising the rate of information transfer about the sampled distribution, viewing the sampler as a noisy communication channel. More theoretical work by Gelman *et al.* suggests an acceptance rate of 0.44 for unidimensional systems, dropping to 0.23 for dimensionalities greater than 5 (Gelman *et al.*, 1996, 2014; Roberts *et al.*, 1997); this may be achieved for Gaussian proposal distributions and approximately normal

posteriors on independent and identically distributed variables by choosing a jumping kernel which has a covariance matrix scaled relative to the target distribution by a factor of $2.38/\sqrt{d}$ (for dimensionality d). Alternatively, for highly non-Gaussian systems, the expected squared jump distance for the Markov chain can be maximised (Pasarica & Gelman, 2010). Peltola *et al.* take this last approach, deriving the optimisation target for the binary variables in their sparse linear regression problem. On simulated and real data, our sampler never approaches optimal acceptance rates (presumably because of the high correlations induced over samples by the Gibbs structure over columns), and it suffices to maximise the number of edges changed per sample.

In total, we divide sampling into five phases.

1. Sample from the full model to allow all parameters to converge without the sparsity constraints. Set $c = 0.5$, $\boldsymbol{\Sigma}_{\log\sigma} = \boldsymbol{I}$ and $m_{ij} = 0.5$.

2. Introduce the MH sampler on $\boldsymbol{Z}$, moving one edge at a time (for example, by setting the geometric distribution parameter l to 0.999). This allows the sampler to move towards a likely model structure. Set $\boldsymbol{\Sigma}_{\log\sigma}$ to match the covariance of the sampled values of $\boldsymbol{\sigma}_{12}$ about the posterior mean, and set the scaling for its MH kernel $c = 2.38/\sqrt{q}$. Periodically update the covariance structure for the sampler on $\boldsymbol{\Sigma}_{\log\sigma}$ and adjust c with proportional increases or decreases to achieve an acceptance rate for samples in $\log\boldsymbol{\sigma}$ of about 0.23.

3. Start adjustment of l. A bracketing system is used to identify the optimal value, which maximises the number of edges accepted per sample. Periodically update the estimate of edge inclusion probabilities $\boldsymbol{M}$, using the history of samples of $\boldsymbol{Z}$. Fix $\boldsymbol{\Sigma}_{\log\sigma}$ and c in this phase.

4. Last phase of the warm-up. Fix l. Continue adjustment of edge inclusion probabilities $\boldsymbol{M}$ and the covariance of $\log\boldsymbol{\sigma}$, $\boldsymbol{\Sigma}_{\log\sigma}$.

5. Sampling phase. Set all sampling hyperparameters ($\boldsymbol{M}$, l, $\boldsymbol{\Sigma}_{\log\sigma}$ and c). Only these samples are used for inference.

A.3 SAMPLING ALGORITHM

Algorithm 2: MCMC SAMPLING ALGORITHM for Hierarchical Inference of Posterior Precisions in OSL (HIPPO).

1. Draw top-level probabilities of edge inclusion from a Beta distribution, incorporating the relative proportions of edges present or absent in the model.

$$a|\boldsymbol{Z}, a_\pi, b_\pi \sim \text{Beta}\left(a_\pi + \sum_{i<j} z_{ij}, b_\pi + n_E - \sum_{i<j} z_{ij}\right) \tag{23}$$

where $n_E = \frac{p(p-1)}{2}$ is the total number of edges.

2. Sample auxiliary variable σ_η as a variance parameter for edges of strength η_{ij}.

$$\sigma_\eta^2|\boldsymbol{\eta}, A \sim \text{Inv-}\chi^2\left(\sum_{i<j} z_{ij} + 1, \frac{A + \sum_{i<j} z_{ij}\eta_{ij}^2}{1 + \sum_{i<j} z_{ij}}\right) \tag{24}$$

3. Sample auxiliary variable ξ, which takes the form of a posterior from linear regression over all edges.

$$\xi | \{\mathbf{\Omega}_s\}, \boldsymbol{\sigma}, \boldsymbol{\eta} \sim \mathcal{N}\left(\frac{\sum_{i<j} \frac{z_{ij}}{\sigma_{ij}^2} \sum_s \omega_{ij}^s \eta_{ij}}{1 + \sum_{i<j} \frac{z_{ij} N \eta_{ij}^2}{\sigma_{ij}^2}}, \frac{1}{1 + \sum_{i<j} \frac{z_{ij} N \eta_{ij}^2}{\sigma_{ij}^2}} \right) \tag{25}$$

4. Sample the regularisation on the diagonal for each subject, which is Gamma distributed under its conjugate prior.

$$\lambda_s | \mathbf{\Omega}_s \sim \mathrm{Ga}\left(\frac{1}{3} + p, \sum_i \frac{\omega_{ii}^s}{2} \right) \tag{26}$$

5. Reform $\chi = |\xi| \sigma_\eta$

6. Choose column j at random from $\{1 \ldots p\}$, without replacement.

   (a) Re-arrange rows and columns of each set of matrices, so that column j is at the end.

   (b) Compute the principal minor $\mathbf{\Omega}_{11}^s$, its inverse

   $$\mathbf{\Omega}_{11}^{s\ -1} = \mathbf{\Sigma}_{11}^s - \mathbf{\Sigma}_{12}^s \mathbf{\Sigma}_{12}^{s\ \dagger} / \Sigma_{22}^s, \tag{27}$$

   the diagonal matrix of inverse-variances $D_\tau = \mathrm{diag}\left(\sigma_{ij}^{-2}\right)$ and, for those edges in the model,

   $$\mathbf{\Upsilon}^s = (s_{22} + \lambda^s)\, \mathbf{\Omega}_{11}^{s\ -1} \tag{28}$$

   $$\mathbf{\Delta}_+^s = \left(\mathbf{\Upsilon}_+^s + D_\tau^+ \right)^{-1}. \tag{29}$$

   (c) Sample edge inclusion variables, $\boldsymbol{\gamma} = z_{12}$, drawing values of σ_{ij}, η_{ij} from their priors (for proposed additional edges) when necessary to compute the acceptance ratio'
   Generate a proposal move $(\boldsymbol{\gamma}^\star, \boldsymbol{\zeta}^\star, \boldsymbol{\zeta})$ using proposal distribution

   $$q_1\left(\boldsymbol{\gamma}^\star, \boldsymbol{\zeta}^\star, \boldsymbol{\zeta}, k | \boldsymbol{\gamma}\right) = p(k)\, q_1\left(\boldsymbol{\gamma}^\star | \boldsymbol{\gamma}, \boldsymbol{\zeta}^\star\right) q_1\left(\boldsymbol{\zeta}^\star | \boldsymbol{\gamma}\right) \tag{30}$$

   and accept with probability $a_1(\boldsymbol{\gamma}^\star | \boldsymbol{\zeta}^\star, \boldsymbol{\zeta}, \boldsymbol{\gamma})$ (10). Perform a low-rank update to $\mathbf{\Delta}_+^s$ if necessary.
   If this first proposal is rejected, perform a delayed rejection step, accepting with certainty one of the models formed by applying a subset of elements of $\boldsymbol{\zeta}^\star$ to $\boldsymbol{\gamma}$. The accepted model is drawn from the proposal distribution $q_2(\boldsymbol{\gamma}' | \boldsymbol{\gamma}, \boldsymbol{\zeta}^\star)$ (19).

   (d) Sample contributions to group mean edge strengths, integrating out subject-level strengths.

   $$\boldsymbol{\eta}_{12}^+ | \sigma_\eta, \xi, \{Y_s\}, \boldsymbol{\sigma} \sim \mathcal{N}\left(-\mathbf{\Xi}^+ \sum_s \left(D_\tau^+ \mathbf{\Delta}_+^s s_{12}^+\right)/\xi,\ \mathbf{\Xi}^+/\xi^2 \right) \tag{31}$$

   for $\mathbf{\Xi}^{+-1} = \frac{1}{\chi^2} + N D_\tau^+ - \sum_s \left(D_\tau^+ \mathbf{\Delta}_+^s D_\tau^+ \right)$.

   (e) Reform $\mu_{ij} = \xi \eta_{ij}$.

   (f) Sample substituted variable v^s for each subject.

   $$v^s | Y_s, \lambda_s \sim \mathrm{Ga}\left(\frac{n_s}{2} + 1, \frac{S_{22} + \lambda_s}{2} \right) \tag{32}$$

(g) Sample edge strengths for each subject.

$$\boldsymbol{u}_s | \sim \mathcal{N}\left(\boldsymbol{\Delta}_+^s \left(D_\tau^+ \boldsymbol{\mu}_+ - S_{12}^+\right), \boldsymbol{\Delta}_+^s\right) \tag{33}$$

(h) Undo parametrisations $\boldsymbol{\omega}_{12}^s = \boldsymbol{u}^s$ and $\omega_{22}^s = \nu^s + \boldsymbol{u}^{s\dagger} {\boldsymbol{\Omega}_{11}^s}^{-1} \boldsymbol{u}^s$.

(i) Update the subject covariance matrices in block fashion.

$$\boldsymbol{\Sigma}_{12}^s = -{\boldsymbol{\Omega}_{11}^s}^{-1} \boldsymbol{u}^s / \nu^s \tag{34}$$

$$\Sigma_{22}^s = 1/\nu^s \tag{35}$$

$$\boldsymbol{\Sigma}_{11}^s = {\boldsymbol{\Omega}_{11}^s}^{-1} + \left({\boldsymbol{\Omega}_{11}^s}^{-1} \boldsymbol{u}^s\right)\left({\boldsymbol{\Omega}_{11}^s}^{-1} \boldsymbol{u}^s\right)^\dagger / \nu^s \tag{36}$$

(j) Sample the subject-level variance, for those edges for which $z_{ij} = 1$, using an MH sampler with a Gaussian proposal distribution on $\log \boldsymbol{\sigma}_{12}$ to draw from $p\left(\boldsymbol{\sigma}_{12} | \chi, \boldsymbol{\gamma}, \boldsymbol{\mu}_{12}, \{Y_s\}\right)$.

(k) Return to loop over columns until complete.

A.4 INITIALISATION AND ASSESSMENT OF CONVERGENCE

It is good practice in Bayesian computation to run several MCMC chains from different starting points, then assess the quality of the inference through a process of calculating heuristics and inspecting the chains (Gelman *et al.*, 2014). Common heuristics include assessing the effective number of samples in the chains n_{eff} (using the same formula that we employ to estimate temporal degrees of freedom in our data, equation 5), and requiring that the Gelman-Rubin scale reduction statistic $\hat{R}$ falls below 1.1 for all parameters (Gelman & Rubin, 1992). However, both of these estimates are only valid for approximately normally-distributed parameters. Furthermore, we have discussed in section A.2.1 the impossibility of completely exploring the parameter space of the sparse model. The Metropolis sampler is able to converge very rapidly to plausible models, but in our experience tends to get stuck in local minima.

We therefore take a two-pronged, pragmatic approach. Firstly, we initialise the sampler using a quick exploration of the target space. We run a large number of model chains (say 10) for a short amount of time (say 5000 samples). Typically, one chain will find a set of models with consistently higher probabilities than the other chains. We seed the full inference run using a subset of models from this first bout as initialisation points for the network structure, picking model structures using an importance sampling algorithm on the exploration samples. This enables us to initialise inference from a region of high probability.

Secondly, we perform inference using several sampling chains (perhaps 4), for as long as we can afford computationally. Afterwards, we inspect the chains visually, and compute n_{eff} and $\hat{R}$ for the non-binary variables. If the chains have explored very different parts of the probability space, we can either run the simulations for longer, restart the inference with new initialisation points, or make do with a poor characterisation of the posterior. This method is similar in style to the practical attitude taken in the variable selection literature, searching and averaging over a subset of plausible models (George & McCulloch, 1993, 1997; Raftery *et al.*, 1997). While not characterising in full the highly multi-modal posteriors, if it is able to improve on the performance of previous algorithms for covariance modelling and provide interpretable inference, then we feel that our approach is justified.

A.5 POSTERIOR SUMMARIES

With the caveats mentioned above held in mind, the set of samples from the MCMC chains can provide (approximately) a complete description of the posterior. Typically, the posterior over particular variables can be summarised with the mean and variance of the relevant samples. Two small subtleties pertain. Firstly, we summarise the graphical model structure not with the mode of the distribution (the highest

probability model), but with the median probability model (constructed by taking the mean of Z across all samples), for which there is greater theoretical support (Barbieri & Berger, 2004; Guan & Stephens, 2011). Secondly, we have drawn samples of the precision and covariance matrices for each subject, but we are more interested in the correlation and partial correlation matrices as expressions of functional connectivity. Following Marrelec *et al.* (2006), we simply rescale each sample, computing partial correlations as

$$\rho_{ij} = -\frac{\omega_{ij}}{\sqrt{\omega_{ii}\omega_{jj}}} \tag{37}$$

and construct samples of population-level effects by averaging the partial correlation matrices across subjects.

B Conditional distributions

We derive the conditional distributions needed for the Gibbs draws in the MCMC scheme.

From the joint distribution (8), we start by extracting the conditional distribution for all variables grouped into columns, taking the last column without loss of generality. We perform the variable substitutions in equation (5) in the main text

$$
\begin{aligned}
(\boldsymbol{u}^s,\, v^s) &= \left(\boldsymbol{\omega}_{12}^s,\, \omega_{22}^s - \boldsymbol{\omega}_{12}^{s\,\dagger} \boldsymbol{\Omega}_{11}^{s\,-1} \boldsymbol{\omega}_{12}^s\right) \\
\boldsymbol{\Upsilon}^s &= (s_{22} + \lambda^s)\, \boldsymbol{\Omega}_{11}^{s\,-1} ,
\end{aligned} \tag{38}
$$

to yield

$$
\begin{aligned}
p\left(\boldsymbol{\gamma}, \boldsymbol{\mu}_{12}, \boldsymbol{\sigma}_{12}, \boldsymbol{u}^s \middle| \{Y_s\}, -\right) \propto \prod_s \Bigg\{ & \frac{1}{(2\pi)^{n_s/2}} e^{-\frac{1}{2}\boldsymbol{u}_+^{s\dagger} \boldsymbol{\Upsilon}_+^s \boldsymbol{u}_+^s - S_{12}^{+\dagger} \boldsymbol{u}_+^s} \frac{|D_\tau^+|^{1/2}}{(2\pi)^{|\gamma_+|/2}} e^{-\frac{1}{2}\left(\boldsymbol{u}_+^s - \boldsymbol{\mu}_+\right)^\dagger D_\tau^+ \left(\boldsymbol{u}_+^s - \boldsymbol{\mu}_+\right)} \\
& \times e^{-\frac{1}{2}\boldsymbol{u}_-^{s\dagger} \boldsymbol{\Upsilon}_-^s \boldsymbol{u}_-^s - S_{12}^{-\dagger} \boldsymbol{u}_-^s} \delta\left(\boldsymbol{u}_-^s\right) \Bigg\} \\
& \times a^{|\gamma_+|} (1-a)^{|\gamma_-|}\, \pi\left(\boldsymbol{\mu}_{12}\right) \pi\left(\boldsymbol{\sigma}_{12}\right) ,
\end{aligned} \tag{39}
$$

where $\boldsymbol{\gamma} = \boldsymbol{z}_{12}$, the edge inclusion variables for the column; symbols $+$ and $-$ denote the subsets of variables within the column that are included or excluded, respectively, in the model; $|\gamma_+|$ is the number of included variables in the column; $D_\tau = \operatorname{diag}\left(\boldsymbol{\sigma}_{12}^{-2}\right)$; and $\pi(x)$ indicates the prior on variable x. Complete the square to find

$$
\begin{aligned}
p\left(\boldsymbol{\gamma}, \boldsymbol{\mu}_{12}, \boldsymbol{\sigma}_{12}, \boldsymbol{u} \middle| \{Y_s\}, -\right) \propto & \frac{1}{(2\pi)^{\sum_s n_s/2}} \frac{1}{(2\pi)^{N|\gamma_+|/2}} |D_\tau^+|^{N/2} a^{|\gamma_+|} (1-a)^{|\gamma_-|} e^{-\frac{N}{2}\boldsymbol{\mu}_+^\dagger D_\tau^+ \boldsymbol{\mu}_+} \\
& \times \prod_s \left\{ e^{-\frac{1}{2}\boldsymbol{u}_+^{s\dagger} \boldsymbol{\Delta}_+^{s\,-1} \boldsymbol{u}_+^s + \boldsymbol{\rho}_+^{s\dagger} \boldsymbol{u}_+^s} \right\} \pi\left(\boldsymbol{\mu}_{12}\right) \pi\left(\boldsymbol{\sigma}_{12}\right) ,
\end{aligned}
$$

where $\boldsymbol{\rho}_+^s = \left(D_\tau^+ \boldsymbol{\mu}_+ - S_{12}^+\right)$ and $\boldsymbol{\Delta}_+^s = \left(\boldsymbol{\Upsilon}_+^s + D_\tau^+\right)^{-1}$.

This immediately gives the conditional for $\boldsymbol{u}$,

$$
\boldsymbol{u}_+^s \sim \mathcal{N}\left(\boldsymbol{\Delta}_+^s \left(D_\tau^+ \boldsymbol{\mu}_+ - S_{12}^+\right), \boldsymbol{\Delta}_+^s\right) \tag{40}
$$

$$
\boldsymbol{u}_-^s = 0 . \tag{41}
$$

The subject-level variables $\boldsymbol{u}$ can be marginalised over,

$$
\begin{aligned}
p\left(\boldsymbol{\gamma}, \boldsymbol{\mu}_{12}, \boldsymbol{\sigma}_{12} \middle| \{Y_s\}, -\right) \propto & \frac{1}{(2\pi)^{\sum_s n_s/2}} \frac{1}{(2\pi)^{N|\gamma_+|/2}} |D_\tau^+|^{N/2} a^{|\gamma_+|} (1-a)^{|\gamma_-|} e^{-\frac{N}{2}\boldsymbol{\mu}_+^\dagger D_\tau^+ \boldsymbol{\mu}_+} \\
& \times \prod_s \left\{ \int \mathrm{d}\boldsymbol{u}_s^+\, e^{-\frac{1}{2}\boldsymbol{u}_+^{s\dagger} \boldsymbol{\Delta}_+^{s\,-1} \boldsymbol{u}_+^s + \boldsymbol{\rho}_+^{s\dagger} \boldsymbol{u}_+^s} \right\} \pi\left(\boldsymbol{\mu}_{12}\right) \pi\left(\boldsymbol{\sigma}_{12}\right) \\
\propto & \frac{1}{(2\pi)^{\sum_s n_s/2}} |D_\tau^+|^{N/2} a^{|\gamma_+|} (1-a)^{|\gamma_-|} e^{-\frac{N}{2}\boldsymbol{\mu}_+^\dagger D_\tau^+ \boldsymbol{\mu}_+} \\
& \times \prod_s \left\{ |\boldsymbol{\Delta}_+^s|^{1/2} e^{\frac{1}{2}\boldsymbol{\rho}_+^{s\dagger} \boldsymbol{\Delta}_+^s \boldsymbol{\rho}_+^s} \right\} \pi\left(\boldsymbol{\mu}_{12}\right) \pi\left(\boldsymbol{\sigma}_{12}\right) ,
\end{aligned} \tag{42}
$$

giving the conditional distribution for $\boldsymbol{\mu}_{12}$, marginal over $\boldsymbol{u}_s$,

$$p\left(\boldsymbol{\mu}_{12}|\chi,\boldsymbol{\gamma},\boldsymbol{\sigma}_{12},\{Y_s\}\right)\propto\frac{1}{(2\pi\chi^2)^{\frac{|\gamma_-|}{2}}}e^{-\frac{1}{2}\boldsymbol{\mu}_-\boldsymbol{\mu}_-/\chi^2}\frac{1}{(2\pi\chi^2)^{\frac{|\gamma_+|}{2}}}e^{-\frac{1}{2}\boldsymbol{\mu}_+\boldsymbol{\Xi}^{-1}\boldsymbol{\mu}_+-\boldsymbol{\epsilon}_+^\dagger\boldsymbol{\mu}_+} \tag{43}$$

$$\begin{aligned}p\left(\boldsymbol{\eta}|\xi,\sigma_\eta,\boldsymbol{\gamma},\boldsymbol{\sigma}_{12},\{Y_s\}\right)&\propto\xi^q\,p\left(\xi\boldsymbol{\eta}|\chi,\boldsymbol{\gamma},\boldsymbol{\sigma}_{12},\{Y_s\}\right)\\&\propto\xi^q\frac{1}{(2\pi\chi^2)^{\frac{|\gamma_-|}{2}}}e^{-\frac{1}{2}\xi^2\boldsymbol{\eta}_-\boldsymbol{\eta}_-/\chi^2}\frac{1}{(2\pi\chi^2)^{\frac{|\gamma_+|}{2}}}e^{-\frac{1}{2}\xi^2\boldsymbol{\eta}_+\boldsymbol{\Xi}^{-1}\boldsymbol{\eta}_+-\xi\boldsymbol{\epsilon}_+^\dagger\boldsymbol{\eta}_+},\end{aligned} \tag{44}$$

where $q=p-1$, the number of edges in a column, $\boldsymbol{\Xi}=\left(\frac{1}{\chi^2}+ND_\tau^+-\sum_s D_\tau^+\boldsymbol{\Delta}_+^sD_\tau^+\right)^{-1}$ and $\boldsymbol{\epsilon}_+^\dagger=\sum_s S_{12}^{+\dagger}\boldsymbol{\Delta}_+^sD_\tau^+$.

The conditional distribution for $\boldsymbol{\sigma}_{12}$ can also be drawn out here,

$$\begin{aligned}p\left(\boldsymbol{\sigma}_{12}|\chi,\boldsymbol{\gamma},\boldsymbol{\mu}_{12},\{Y_s\}\right)\propto&\frac{1}{(2\pi)^{\sum_s n_s/2}}|D_\tau^+|^{N/2}e^{-\frac{N}{2}\boldsymbol{\mu}_+^\dagger D_\tau^+\boldsymbol{\mu}_+}\\&\times\prod_s\left\{|\boldsymbol{\Delta}_+^s|^{1/2}e^{\frac{1}{2}\boldsymbol{\rho}_+^{s\dagger}\boldsymbol{\Delta}_+^s\boldsymbol{\rho}_+^s}\right\}\\&\times\prod_i\left\{\frac{1}{\sigma_{i.}\left(2\pi s_\sigma^2\right)^{\frac{1}{2}}}e^{-\frac{(\log\sigma_{i.}-\log m_\sigma)^2}{2s_\sigma^2}}\right\}.\end{aligned} \tag{45}$$

For the edge inclusion variables, we can further integrate out $\boldsymbol{\mu}_{12}$, in the same manner,

$$\begin{aligned}p\left(\boldsymbol{\gamma}|\{Y_s\},\chi,\boldsymbol{\sigma}_{12},a\right)\propto&\frac{1}{(2\pi)^{\sum_s n_s/2}}|D_\tau^+|^{N/2}a^{|\gamma_+|}(1-a)^{|\gamma_-|}\prod_s|\boldsymbol{\Delta}_+^s|^{1/2}\\&\times\int\mathrm{d}\boldsymbol{\mu}_{12}\,e^{-\frac{N}{2}\boldsymbol{\mu}_+^\dagger D_\tau^+\boldsymbol{\mu}_+}\prod_s\left\{e^{\frac{1}{2}\boldsymbol{\rho}_+^{s\dagger}\boldsymbol{\Delta}_+^s\boldsymbol{\rho}_+^s}\right\}\frac{1}{(2\pi\chi^2)^{\frac{q}{2}}}e^{-\mu_{12}^2/2\chi^2}\\\propto&\frac{1}{(2\pi)^{\sum_s n_s/2}}|D_\tau^+|^{N/2}a^{|\gamma_+|}(1-a)^{|\gamma_-|}\prod_s\left\{|\boldsymbol{\Delta}_+^s|^{1/2}e^{\frac{1}{2}S_{12}^{+\dagger}\boldsymbol{\Delta}_+^sS_{12}^+}\right\}\\&\times\int\mathrm{d}\boldsymbol{\mu}_+\,e^{-\frac{1}{2}\boldsymbol{\mu}_+^\dagger\boldsymbol{\Xi}^{-1}\boldsymbol{\mu}_+-\sum_s S_{12}^{+\dagger}\boldsymbol{\Delta}_+^sD_\tau^+\boldsymbol{\mu}_+}\frac{1}{(2\pi\chi^2)^{\frac{|\gamma_+|}{2}}}\\\propto&\frac{1}{(2\pi)^{\sum_s n_s/2}}|D_\tau^+|^{N/2}a^{|\gamma_+|}(1-a)^{|\gamma_-|}\\&\times\prod_s\left\{|\boldsymbol{\Delta}_+^s|^{1/2}e^{\frac{1}{2}S_{12}^{+\dagger}\boldsymbol{\Delta}_+^sS_{12}^+}\right\}\frac{|\boldsymbol{\Xi}|^{1/2}}{\chi^{2^{|\gamma_+|/2}}}e^{\frac{1}{2}\boldsymbol{\epsilon}_+^\dagger\boldsymbol{\Xi}\boldsymbol{\epsilon}_+}.\end{aligned} \tag{46}$$

C Characterisation of the hierarchical models

C.1 MODELLING VARIABILITY

Hierarchical Bayesian models express the connectivity in each individual within the context of the wider group or population, explicitly modelling the variability in connection strengths across subjects. To illustrate this, figure 1 shows the posterior mean group-average partial correlation networks for our MEG beta-band data, together with the posterior mean of the standard deviation in connection strength over subjects. In general, stronger connections also tend to be more variable over subjects (using inferences from the weakly sparse Hierarchical Inference of Posterior Precisions in OSL (HIPPO) model, the correlation between group mean connection strength and the standard deviation across subjects is 0.8). In our dataset, the most variable connection strength is that between the left and right motor cortices, marked by an arrow in the figure. Table 1 indexes the ROI locations in the figure's network matrix plots.

The heritability analyses we report with the MEG data were performed on correlation, rather than partial correlation, matrices, as discussed in the main text (although the main hierarchical network modelling and inference is always built on the inverse covariance). Figure 2 shows the posterior mean estimates used for these analyses, giving the connections within their anatomical context.

C.2 CAPTURING UNCERTAINTY

The Bayesian models produce a posterior distribution for each subject's connectivity strengths, summarised by the set of MCMC samples, that could be used to make inference on, for example, differences in connectivity between groups of subjects. Figure 9 in the main text shows that, on the simulated datasets, the hierarchical models tend to be conservative in their posteriors: the 90% highest density interval contains at least 90% of the true edge strengths. In neuroimaging, where control of false positives is the reigning dictum, erring on the side of caution in this way is acceptable. The most accurate density intervals occur for simulation 6, which was designed to mimic the structure of the prior.

The Bayesian models also describe the uncertainty in the presence of connections in the underlying network. In the strongly sparse HIPPO model, the parameter Z draws posterior samples of this graph structure. The probability of a network connection for the MEG beta-band data is shown in figure 1F. This posterior distribution over the graph could further be used to perform inference on graph-theoretic measures that summarise the network properties. The weakly sparse model does not explicitly infer the network structure, but the posterior samples of the mean partial correlation matrix give the probability that any connection strength is greater (or less than) zero. This is shown in figure 1E.

C.3 OVERWHELMING THE PRIOR WITH DATA

The inference of partial correlation network matrices for our fMRI dataset with the sparse HIPPO model gives one surprising result. The posterior probabilities for the presence of network connections are over 99% on each edge. We suggest that the quantity of data in an hour's total recording time overwhelms the sparse prior. The data are sufficient to provide evidence for connectivity between all nodes, even if this connectivity is very weak in some cases. (See also the broader discussion on this point in Smith & Nichols, 2018.) Further support for this suggestion comes from the individual subjects' networks, which are hardly changed under the hierarchical models from the conventional estimates; this is particularly apparent in comparison to the adjustment made to the MEG networks, shown in figure 4.

We illustrate this effect with a simulation. Using a small, simulated, 5-node Gaussian network in which most of the connections are very small, but non-zero, we perform inference using the sparse HIPPO model on a very short (and therefore noisy) dataset, and for a very long dataset. Figure 5 shows the true network that generated the data, the posterior probability of connections from 15 data points in

5 subjects, and the connection probabilities from 1000 data points from 25 subjects. With limited data, the sparse HIPPO prior tends to force most of the weak connections to zero: the connection probabilities are very low. However, in the larger dataset, the likelihood completely dominates the prior: posterior connection probabilities are 1 on each network edge.

C.4 COMPUTATIONAL EFFICIENCY

Both the weakly sparse hierarchical model and the strongly sparse model have a computational complexity that scales linearly with the number of subjects, and is roughly quartic in the number of network nodes, on a per-sample basis.[1] (Larger models may require more samples to converge.) These scalings are illustrated in figure 6. Sparsity in the model reduces the dimensions of the necessary Cholesky factorisations, which enables the fully-sparse model to outperform the weakly sparse model as the network size increases in figure 6B.

Both methods improve upon the complexity of the best (single-subject) G-Wishart algorithms (the existing, non-hierarchical, sparse Bayesian precision model), which show quintic scaling with the number of nodes (Hinne *et al.*, 2015). A comparison of the speed of inference using our hierarchical approach, a single-subject version of the prior (equation 25 in the main text) and Hinne *et al.*'s G-Wishart sampler is given in table 2, together with inference times for the Sparse Group Gaussian Graphical Model (SGGGM) algorithm. Exploration of the sparse model space is slow using the strongly sparse hierarchical prior—but still faster than the G-Wishart model, which becomes infeasible to run, even for a single subject, on networks with more than 50 nodes. The hierarchical models, and the single-subject version of the HIPPO model, can draw samples within acceptable time-frames even for networks of 200 nodes (table 3). SGGGM, on the other hand, is quick to run, but searching the parameter space can be slow, as some hyper-parameter choices can require thousands of iterations of the algorithm before convergence to a solution. On the large 200-node dataset used in table 3, the optimal hyper-parameter set took 24 hours to identify on cross-validated data using solvers in Matlab.

[1] The main computational burden in each sample is the Cholesky decomposition of $p\,(N+1)$ square matrices of size $(p-1)$ (for each of N subjects, we update each column in turn within a single sample), and a series of rank-1 updates to these decompositions

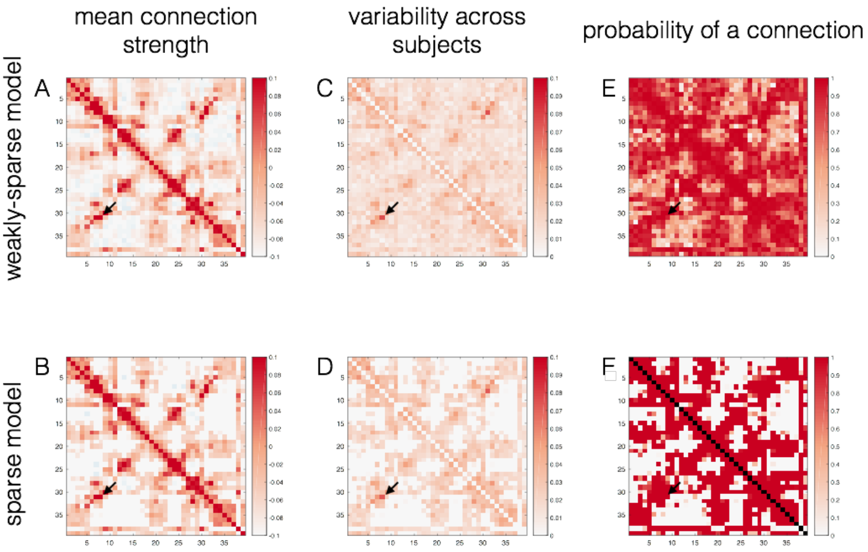

Figure 1: PARTIAL CORRELATION NETWORKS AND BETWEEN-SUBJECT VARIATION FOR THE MEG BETA BAND. A and B, grand mean partial correlation networks for the MEG beta-band (13–30 Hz), computed using the sparse hierarchical model HIPPO (top) and the weakly sparse model (bottom). C and D, standard deviation in partial correlation strength over subjects for each model. The greatest variability over subjects is for the connection between left and right motor cortices, marked with an arrow. E and F, indications of the presence of the underlying edge in the brain network. E gives the posterior probability under the weakly sparse model that the mean connectivity is greater than zero. F gives the posterior probability of edge presence using the inferred parameter Z in the sparse model. Images A–D show posterior means of the relevant parameters. The locations of each ROI are indexed in table 1.

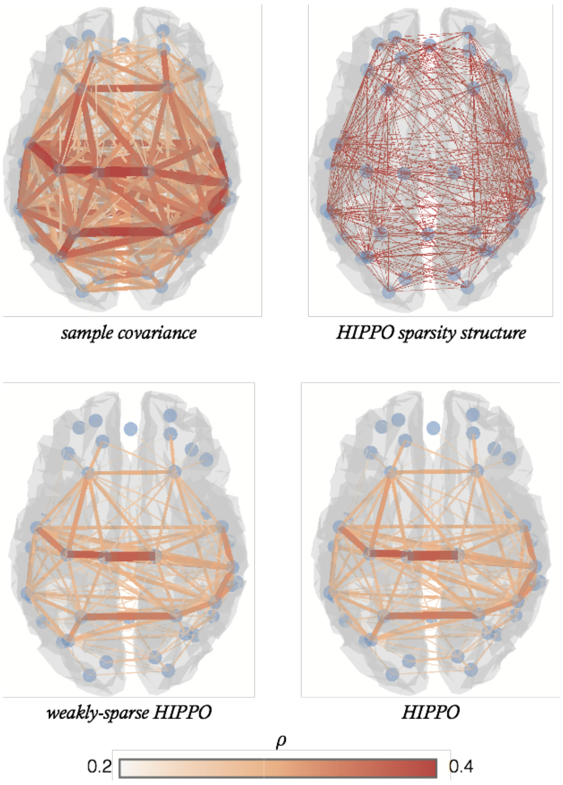

Figure 2: GROUP AVERAGE CORRELATION NETWORKS FOR THE MEG BETA BAND. Group average functional correlation networks in the MEG beta-band (13–30 Hz), computed using three estimation methods: the sample correlation matrix (the same result as used in Colclough *et al.*, 2017; top left), the hierarchical model HIPPO (bottom right) and the weakly sparse version of HIPPO (bottom left). The colour scale, ρ, indicates the strength of these correlations (using the posterior mean). Also shown (top right) is the median probability model of direct connections in the group partial correlation matrix, computed using HIPPO—these are the *direct* functional connections inferred with $p > 0.5$ by the model.

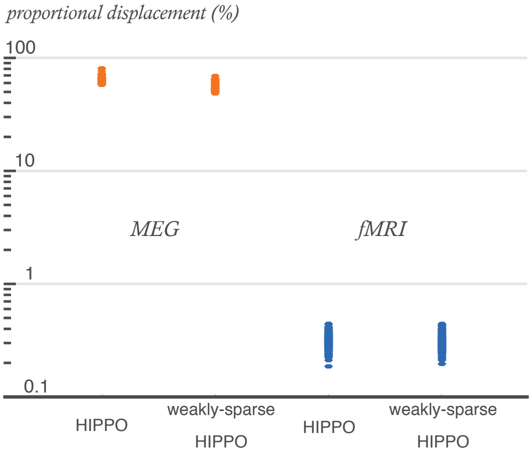

Figure 4: DISPLACEMENT OF NETWORK MATRICES FROM THEIR UNREGULARISED FORMS after estimation with the hierarchical model. The RMS distance between partial correlation matrices, inferred with the strongly and weakly sparse hierarchical models, and the standard Tikhonov-regularised estimates, is displayed as a percentage fraction (on a log scale) of the mean connection strength in each subject. Each dot represents a different subject. Much more adjustment was made to the MEG matrices than to the fMRI networks.

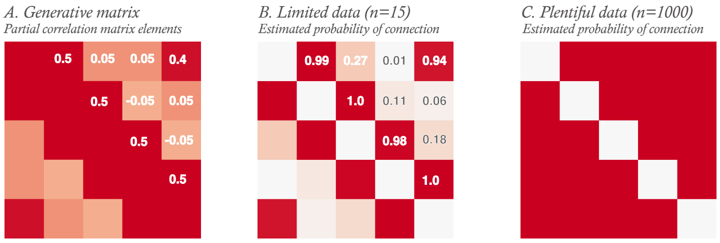

Figure 5: SUFFICIENT DATA CAN OVERWHELM SPARSE PRIORS. A. Partial correlation matrix used to generate two datasets: one with 5 subjects, each with 15 samples, and one with 25 subjects, each with 1000 samples. B. Posterior connection probabilities from the strongly sparse HIPPO model in the smaller dataset, where the sparse prior encourages the belief that the weak edges are in fact no present in the graph. C. Posterior connection probabilities from inference on the larger dataset, where the data are sufficiently strong that the model infers a connection on each edge with probability 1, even for weak correlations.

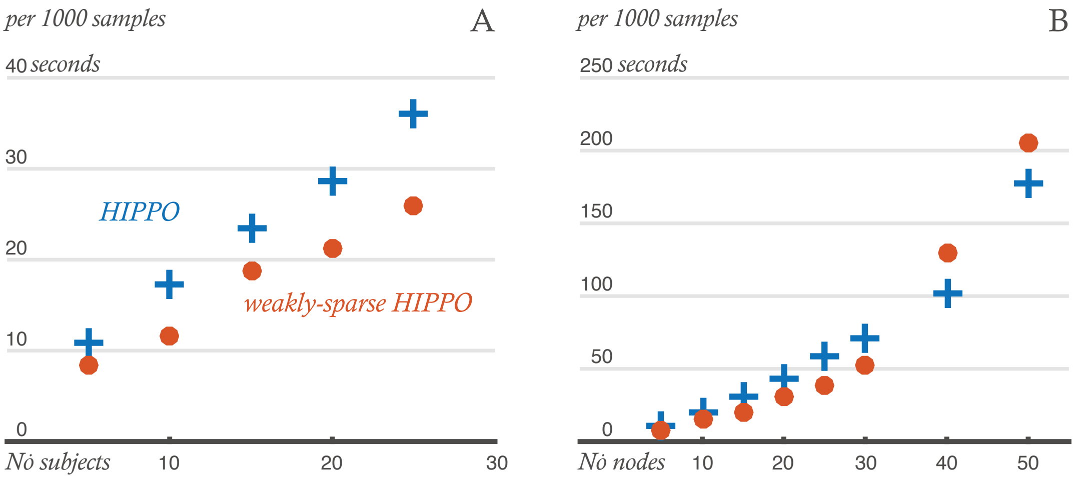

Figure 6: SCALING OF COMPUTATIONAL COST WITH CHANGING MODEL DIMENSION. The time for 1000 samples to be drawn using the weakly and strongly sparse hierarchical model (HIPPO) as the dimensions of the problem are altered. The scaling is linear as the number of subjects are increased (A) and can be quartic as the number of network nodes increases (B). Estimates are the average of 10 runs on 5-node simulation 2 and 5 subjects of simulation 9, respectively.

Table 1: INDEX OF ROI NUMBERS.

ROI number	ROI location
1, 2, 3, 4, 5	Left Frontal Lobe
6	Left Somatosensory Cortex
7, 8	Left Motor Cortex
9, 10, 11	Left Parietal Cortex
12, 13	Left Visual Cortex
14, 15	Left Occipital Lobe
16, 17, 18	Left Temporal Lobe
19, 20, 21	Right Temporal Lobe
22, 23	Right Occipital Lobe
24, 25	Right Visual Cortex
26, 27, 28, 29, 30	Right Parietal Lobe
31	Right Motor Cortex
32	Right Somatosensory Cortex
33, 34, 35, 36, 37	Right Frontal Lobe
38	Posterior Cingulate Cortex
39	Medial Frontal Cortex

that scale with the number of edges proposed for addition or removal in the MH step. The Cholesky decomposition has $\mathcal{O}\left(n^3\right)$ complexity in general for square matrices of size n, although this can be reduced for sparse matrix computations.

Table 2: COMPARATIVE COMPUTATIONAL COST OF SPARSE BAYESIAN MODELS AND SGGGM ON A 30-NODE PROBLEM. The time taken to draw 1000 MCMC samples, or to move through 1000 different model structures, for the sparse Bayesian samplers tested on simulated data. Of these, only the weakly sparse and strongly sparse HIPPO were fitted to all subjects separately and simultaneously, the other two were fitted to the entire concatenated dataset. Also shown is the time for the SGGGM model to converge, using one setting of its hyper-parameters. Inference times for SGGGM will be affected by the speed at which optimal hyper-parameters can be discovered. Timings are given using an average of ten runs on dataset 10, which has 25 subjects, 30 nodes and 100 time points, using a MacBook Pro with 2.8 GHZ processor and 16 GB of RAM. All times are given in seconds.

25 subjects, 30 nodes	Strongly sparse HIPPO	Weakly sparse HIPPO	Single-subject HIPPO	G-Wishart	SGGGM
Time for 1000 samples *(seconds)*	250	180	11	6600	
Time for 1000 samples per subject	10	7	11	6600	
Time for 1000 models	4200	–	12	6600	
Time for 1000 models per subject	170	–	12	6600	
Time for 1 iteration of the solver	–	–	–	–	0.7
Time for convergence using the optimal hyper-parameter set	–	–	–	–	65

Table 3: COMPARATIVE COMPUTATIONAL COST OF SPARSE BAYESIAN MODELS AND SGGGM ON A 200-NODE PROBLEM. The table gives the time taken to draw 1000 MCMC samples, or to move through 1000 different model structures, for the sparse Bayesian samplers tested on simulated data. Of these, only the weakly sparse and strongly sparse HIPPO were fitted to all subjects separately and simultaneously, the other two were fitted to the entire concatenated dataset. No times are given for the G-Wishart model, as not even 10 samples were drawn in 12 hours. Also shown is the time for the SGGGM model to converge, using one setting of its hyper-parameters. Inference times for SGGGM will be affected by the speed at which optimal hyper-parameters can be discovered. All timings are given for one run on an example sparse dataset built in the same manner as dataset 5, but using a 4×4 re-tiling of the adjacency matrix. This dataset has 25 subjects, 200 nodes, 10 000 time points, and a sparsity of 0.97. The strongly sparse HIPPO model can exploit this sparsity to sample more rapidly than the weakly sparse model, which assumes a full network graph. Times were measured using a MacBook Pro with 2.8 GHZ processor and 16 GB of RAM. All times are given in seconds.

25 subjects, 200 nodes	Strongly sparse HIPPO	Weakly sparse HIPPO	Single-subject HIPPO	G-Wishart	SGGGM
Time for 1000 samples *(seconds)*	8725	27820	380	$>10^6$	
Time for 1000 samples per subject	350	1110	380	$>10^6$	
Time for 1000 models	218125	–	425	$>10^6$	
Time for 1000 models per subject	8725	–	425	$>10^6$	
Time for 1 iteration of the solver	–	–	–	–	0.8
Time for convergence using the optimal hyper-parameter set	–	–	–	–	65

References

BARBIERI, M.M. & BERGER, J.O. (2004) 'Optimal predictive model selection.' *Annals of Statistics*, **32**, 81–94.

BECKMANN, C.F. *et al.* (2009) 'Group comparison of resting-state fMRI data using multi-subject ICA and dual regression.' In *Proceedings of the 15th annual meeting of the Organization for Human Brain Mapping*. OHBM, San Francisco, CA.

CALHOUN, V.D. *et al.* (2001) 'A method for making group inferences from functional MRI data using independent component analysis.' *Human Brain Mapping*, **14**, 140–151.

CARVALHO, C.M., POLSON, N.G. & SCOTT, J.G. (2010) 'The horseshoe estimator for sparse signals.' *Biometrika*, **97**(2), 465–480.

CHAPPELL, M.A. *et al.* (2009) 'Variational bayesian inference for a nonlinear forward model.' IEEE *Transactions on Signal Processing*, **57**, 223–236.

CLARKE, M.R.B. (1981) 'A Givens algorithm for moving from one linear model to another without going back to the data.' *Journal of the Royal Statistical Society C*, **30**(2), 198–203.

COLCLOUGH, G.L. *et al.* (2017) 'The heritability of multi-modal connectivity in human brain activity.' *eLife*, **6**, e20 178.

VAN DYK, D.A. & PARK, T. (2008) 'Partially collapsed Gibbs samplers: theory and methods.' *Journal of the American Statistical Association*, **103**(482), 790–796.

FILIPPINI, N. *et al.* (2009) 'Distinct patterns of brain activity in young carriers of the APOE-ϵ4 allele.' *Proceedings of the National Academy of Sciences* USA, **106**, 7209–7214.

GELMAN, A. (2006) 'Prior distributions for variance parameters in hierarchical models.' *Bayesian Analysis*, **1**(3), 515–533.

GELMAN, A., ROBERTS, G.O. & GILKS, W.R. (1996) 'Efficient Metropolis jumping rules.' *Bayesian Statistics*, **5**, 599–607.

GELMAN, A. & RUBIN, D.B. (1992) 'Inference from iterative simulation using multiple sequences.' *Statistical Science*, **7**(4), 457–511.

GELMAN, A. *et al.* (2008) 'Using redundant parameterizations to fit hierarchical models.' *Journal of Computational and Graphical Statistics*, **17**(1), 95–122.

——— (2014) *Bayesian Data Analysis*. CRC Press, Taylor & Francis Group, Boca Raton, FL, 3rd edition.

GEORGE, E.I. & MCCULLOCH, R.E. (1993) 'Variable selection via Gibbs sampling.' *Journal of the American Statistical Association*, **88**(423), 881–889.

——— (1997) 'Approaches for Bayesian variable selection.' *Statistica Sinica*, **7**, 339–373.

GREEN, P.J. & MIRA, A. (2001) 'Delayed rejection in reversible jump Metropolis-Hastings.' *Biometrika*, **88**, 1035–1053.

GROVES, A.R. *et al.* (2011) 'Linked independent component analysis for multimodal data fusion.' *NeuroImage*, **54**(3), 2198–2217.

GUAN, Y. & STEPHENS, M. (2011) 'Bayesian variable selection regression for genome-wide association studies and other large-scale probelms.' *Annals of Applied Statistics*, **5**(3), 1780–1815.

HARRISON, S.J. *et al.* (2015) 'Large-scale probabilistic functional modes from resting-state FMRI.' *NeuroImage*, **109**, 217–231.

HINNE, M. *et al.* (2015) 'Bayesian estimation of conditional independence graphs improves functional connectivity estimates.' *PLoS Computational Biology*, p. 10.1371/journal.pcbi.1004534.

LIU, C., RUBIN, D.B. & WU, Y.N. (1998) 'Parameter expansion to accelerate EM: the PX-EM algorithm.' *Biometrika*, **85**(4), 755–770.

MACKAY, D.J.C. (2003) *Information Theory, Inference, and Learning Algorithms.* Cambridge University Press, Cambridge, UK.

MARRELEC, G. *et al.* (2006) 'Partial correlation for functional brain interactivity investigation in functional MRI.' *NeuroImage*, **32**(1), 228–237.

MIRA, A. (2001) 'On Metropolis-Hastings algorithms with delayed rejection.' *Metron*, **59**, 231–241.

PASARICA, C. & GELMAN, A. (2010) 'Adaptively scaling the Metropolis algorithm using expected squared jumped distance.' *Statistica Sinica*, **20**.

PELTOLA, T., MARTTINEN, P. & VEHTARI, A. (2012) 'Metropolis-Hastings algorithm for variable selection in genome-wide association analysis.' *PLoS ONE*, **7**(11), e49 445.

PENNY, W.D., KILNER, J.M. & BLANKENBUR, F. (2007) 'Robust Bayesian general linear models.' *NeuroImage*, **36**, 661–671.

POLSON, N.G. & SCOTT, J.G. (2012) 'On the half-Cauchy prior for a global scale parameter.' *Bayesian Analysis*, **7**(2), 1–16.

RAFTERY, A., MADIGAN, D. & HOETING, J.A. (1997) 'Bayesian model averaging for linear regression models.' *Journal of the American Statistical Association*, **92**(437), 179–191.

ROBERTS, G.O., GELMAN, A. & GILKS, W.R. (1997) 'Weak convergence and optimal scaling of random walk Metropolis algorithms.' *Annals of Applied Probability*, **7**(1), 110–120.

SMITH, D.M. (1989) 'All possible subset regressions using the QR decomposition.' *Journal of the Royal Statistical Society C*, **40**(3), 502–513.

SMITH, S.M. & NICHOLS, T.E. (2018) 'Statistical challenges in "big data" human neuroimaging.' *Neuron*, **97**(2), 263–268.

STORVIK, G. (2011) 'On the flexibility of Metropolis-Hastings acceptance probabilities in auxilliary variable proposal generation.' *Scandinavian Journal of Statistics*, **38**(2), 342–358.

TRIAS, M., VECCHIO, A. & VEITCH, J. (2009) 'Delayed rejection schemes for efficient Markov chain Monte Carlo sampling of multimodal distributions.' *ArXiv*:0904.2207v2.

WANG, H. (2015) 'Scaling it up: stochastic search structure learning in graphical models.' *Bayesian Analysis*, **10**(2), 351–377.

WORSLEY, K.J. *et al.* (1992) 'A three-dimensional statistical analysis for CBF activation studies in human brain.' *Journal of Cerebral Blood Flow and Metabolism*, **12**, 900–918.
